# Association of remnant cholesterol with unhealthy lifestyle and risk of coronary heart disease: a population-based cohort study

**DOI:** 10.1016/j.lanepe.2025.101223

**Published:** 2025-02-07

**Authors:** Mia Ø. Johansen, Signe Vedel-Krogh, Sune F. Nielsen, Shoaib Afzal, George Davey Smith, Børge G. Nordestgaard

**Affiliations:** a Department of Clinical Biochemistry, Copenhagen University Hospital - Herlev and Gentofte, Denmark; b The Copenhagen General Population Study, Copenhagen University Hospital - Herlev and Gentofte, Denmark; c Institute of Clinical Medicine, Faculty of Health and Medical Sciences, University of Copenhagen, Denmark; d MRC Integrative Epidemiology Unit (IEU), Bristol Medical School, University of Bristol, United Kingdom; e Population Health Sciences, Bristol Medical School, University of Bristol, United Kingdom

**Keywords:** Atherosclerotic cardiovascular disease, Cholesterol, Lifestyle, Lipids, Myocardial infarction, Nuclear magnetic resonance spectroscopy, Prospective cohort study, Remnant cholesterol, Risk factors

## Abstract

**Background:**

Unhealthy lifestyle is a major risk factor for coronary heart disease, which may be explained by elevated remnant cholesterol. However, this question remains incompletely clarified. In this study, we aimed to investigate whether elevated remnant cholesterol explains part of the excess risk of myocardial infarction and coronary heart disease in individuals with unhealthy lifestyle.

**Methods:**

We included 104,867 individuals (58,286 women and 46,581 men) from the Copenhagen General Population Study free from coronary heart disease at examination. During a median follow-up of 9.2 years, 2484 developed myocardial infarction and 3570 developed coronary heart disease. To understand explained risk from elevated remnant cholesterol due to unhealthy lifestyle on risk of myocardial infarction and coronary heart disease, we used mediation analyses.

**Findings:**

Current smoking, low physical activity, and low adherence to dietary guidelines were all associated with higher levels of remnant cholesterol. For current smoking, remnant cholesterol explained 15% (95% confidence interval: 9.7%–20%) of the excess risk of myocardial infarction and 16% (11%–21%) of the excess risk of coronary heart disease. Corresponding values for low physical activity were 20% (13%–27%) and 21% (15%–28%), and for low adherence to dietary guidelines 12% (6.6%–18%) and 14% (8.0%–19%), respectively. Results were similar in women and men separately and in analyses where each lifestyle factor were additionally adjusted for the other three lifestyle factors.

**Interpretation:**

Elevated remnant cholesterol explained part of excess myocardial infarction and coronary heart disease in individuals with an unhealthy lifestyle. Clinically, these novel findings underline the importance of both elevated remnant cholesterol and promotion of healthy lifestyle in primary prevention of myocardial infarction and coronary heart disease.

**Funding:**

Independent Research Fund Denmark; Johan Boserup and Lise Boserups Grant; 10.13039/501100000265Medical Research Council.

**Research in context**

**Evidence before this study**

We searched PubMed for published studies on the association of i) “Smoking” [Mesh], “Exercise” [Mesh], “Alcohol Drinking” [Mesh], “Diet” [Mesh], or “Life Style” [Mesh], and ii) “Cholesterol, VLDL” [Mesh], “Lipoproteins, VLDL” [Mesh], “remnant-like particle cholesterol” [Supplementary Concept], “remnant cholesterol”, or “triglyceride-rich lipoprotein cholesterol” with risk of iii) “Coronary Disease” [Mesh], “Myocardial Infarction” [Mesh], or “Myocardial Ischemia” [Mesh] published up to February 27th, 2024. It is well-established that unhealthy lifestyle is a major risk factor for coronary heart disease. However, it is most likely the metabolic changes accompanying unhealthy lifestyle, such as elevated low-density lipoprotein (LDL) cholesterol, elevated remnant cholesterol, hypertension, and diabetes that explain such excess risk. Because elevated remnant cholesterol, like elevated LDL cholesterol, is a causal risk factor for coronary heart disease, it seems biologically plausible that elevated remnant cholesterol could explain part of the excess coronary heart disease in unhealthy lifestyle; however, data are limited on to what extent elevated remnant cholesterol explains such excess risk in individuals with unhealthy lifestyle.

**Added value of this study**

Our results from 104,867 individuals (58,286 women and 46,581 men) from the Copenhagen General Population Study suggest that elevated remnant cholesterol explains a substantial proportion of the excess coronary heart disease in individuals with unhealthy lifestyle. We observed that current smoking, low physical activity, and low adherence to dietary guidelines all were associated with higher levels of remnant cholesterol. For current smoking, remnant cholesterol explained 15% of the excess risk of myocardial infarction and 16% of the excess risk of coronary heart disease. Corresponding values for low physical activity were 20% and 21%, and for low adherence to dietary guidelines 12% and 14%, respectively. Results were similar in women and men separately.

**Implications of all the available evidence**

These novel findings suggest that elevated remnant cholesterol explains a substantial proportion of the excess risk of coronary heart disease in unhealthy lifestyle, and clinically, underline the importance of targeting elevated remnant cholesterol in primary prevention strategies and promoting a healthy lifestyle.

## Introduction

Coronary heart disease remains a leading cause of morbidity and mortality worldwide,^1^ in part explained by an increasing prevalence of unhealthy lifestyle in different parts of the world.^2^, ^3^, ^4^, ^5^, ^6^, ^7^ The importance of promoting a healthy lifestyle in reducing risk of coronary heart disease is well-established and represents a cornerstone in primary prevention strategies.^8^, ^9^, ^10^ However, it is most likely the metabolic changes accompanying unhealthy lifestyle, such as elevated remnant cholesterol, elevated low-density lipoprotein (LDL) cholesterol, hypertension, and diabetes, that explain excess risk of coronary heart disease. Nevertheless, their separate contributions are not completely clarified, which hinders optimal prevention efforts for coronary heart disease in individuals with unhealthy lifestyle.

Accumulation of cholesterol from LDL and remnants in the arterial wall is a central component in the development of atherosclerosis.^11^^,^^12^ Current clinical guidelines primarily address monitoring and correction of LDL cholesterol in the primary prevention of coronary heart disease^8^^,^^9^; however, a substantial residual risk of coronary heart disease remains even after LDL cholesterol is reduced below recommended values.^13^, ^14^, ^15^ Emerging evidence suggests that elevated remnant cholesterol likely explains part of this residual risk.^15^, ^16^, ^17^, ^18^, ^19^ An important driver of elevated remnant cholesterol is high body mass index (BMI)^20^, ^21^, ^22^, ^23^ and likely unhealthy lifestyle.^24^, ^25^, ^26^, ^27^, ^28^, ^29^ Since elevated remnant cholesterol is a causal risk factor for coronary heart disease,^30^^,^^31^ it seems biologically plausible that elevated remnant cholesterol could explain part of excess risk of myocardial infarction and coronary heart disease from unhealthy lifestyle; however, data on this are limited.

We aimed to investigate whether elevated remnant cholesterol explains part of the excess risk of myocardial infarction and coronary heart disease in individuals with unhealthy lifestyle. To do so, we included 104,867 individuals from the Copenhagen General Population Study with baseline information on remnant cholesterol, current smoking status, physical activity, alcohol consumption, and dietary behaviour.

## Methods

This study was conducted according to the Declaration of Helsinki and was approved by a Danish Ethics committee (H-KF-01-144/01) and by Copenhagen University Hospital–Herlev and Gentofte. All participants provided written informed consent.

### Study design and participants

**Fig. 1:**
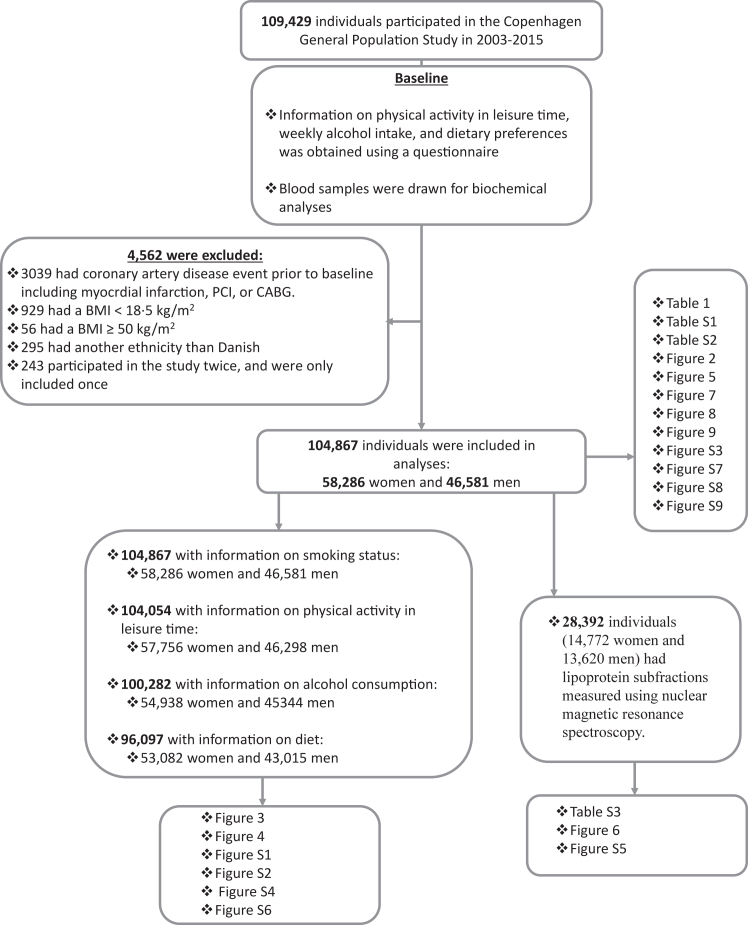
**Flowchart**. This flowchart outlines the study design, including participant selection, and inclusion and exclusion criteria.

The Copenhagen General Population Study (CGPS) is a large, contemporary, prospective cohort study of 109,429 individuals recruited in 2003–2015. Invited individuals were white women and men of Danish descent randomly selected from the national Danish Civil Registration System to represent the general population with age ranging from 20 to 100 years (43% response rate of those invited). When assessing lifestyle factors as risk factors for coronary artery disease, studying a homogeneous population reduces variability due to genetic and environmental differences, making it easier to identify the specific impact of lifestyle factors on health outcomes in the target population. At study entrance, individuals had a physical examination including anthropometric measures, had non-fasting blood samples drawn for biochemical analyses including standard lipid measurements, and filled in a questionnaire about current smoking status, physical activity in leisure time, weekly alcohol intake, and dietary preferences using a short food frequency questionnaire. Among these 109,429 individuals, we included 104,867 (58,286 women and 46,581 men) free from coronary artery disease at examination and with information on smoking status, physical activity, alcohol consumption, and/or dietary adherence, and with BMI ranging from 18.5–50 kg/m^2^ (Fig. 1); we excluded those with BMI <18.5 as such individuals include those with various serious diseases causing weight loss and those with >50 kg/m^2^ as this group is small and represent a very extreme group when it comes to lifestyle. A subgroup of 28,392 (14,772 women and 13,620 men) individuals within the 104,867 individuals had measurements on lipoprotein subfractions measured using nuclear magnetic resonance spectroscopy.

### Lifestyle factors

#### Smoking status

Smoking status was self-reported and categorized into two groups: 1) current smokers, and 2) former/never smokers. We also collected information on daily tobacco consumption (in grams per day), including the number of cigarettes, cheroots, and cigars consumed daily, as well as the weekly amount of pipe tobacco in grams. Additionally, cumulative tobacco consumption was calculated in pack-years, with 1 pack-year defined as daily tobacco use equivalent to smoking 20 cigarettes per day.

#### Physical activity in leisure time

Physical activity during leisure time was self-reported in one of four categories: 1) almost always inactive, or less than 2 h per week of light physical activity (e.g., reading, watching television, going to a movie); 2) two to 4 h per week of light physical activity (e.g., walking, bicycling, light gardening, light gymnastics); 3) more than 4 h of light physical activity, or two to 4 h of vigorous activity per week (e.g., brisk walking or bicycling, heavy gardening, intensive gymnastics); or 4) more than 4 h per week of vigorous physical activity. Based on these responses, low physical activity in leisure time was defined as either less than 4 h of light–intensity activity weekly or less than 2 h of high-intensity activity weekly corresponding to first and second category.

#### Alcohol consumption

Alcohol was self-reported and included information on weekly consumption of bottles of beer, and standard glasses of wine and spirits corresponding to 12 g of pure alcohol per drink. High alcohol consumption was more than 120-g (10 units) alcohol weekly for women and men as recommended by the Danish Health Authority.

#### Adherence to Danish dietary guidelines

Adherence to Danish dietary guidelines were assessed using data from a short food frequency questionnaire that included information on intake of unsaturated fats (soft margarines and vegetable oils) and saturated fats (butter, butter-based blends, and hard margarines) for warm and cold meals, weekly intake of fruit, vegetables, and fish (ranging from almost never to several times daily), weekly intake of sucrose sweetened beverages (ranging from almost never to several times daily), intake of cold meat cuts including sausages and pâtes for open sandwiches (ranging from almost never to several times daily), and weekly intake of fast food (ranging from almost never to several times daily).^32^

We did not have access to information on the remaining Danish dietary recommendations including intake of whole grain foods, intake of low-fat dairy products, intake of low-fat meat and meat products, and intake of water.^32^

Non-adherence to Danish dietary guidelines included preference for saturated fat versus unsaturated fat, low weekly intake of fruit (<3) and/or vegetables (<3), low weekly intake of fish, high intake of sucrose sweetened beverages, high intake of could meat cuts, and high intake of fast food. Dietary preferences in disagreement with current dietary guidelines were classified into three levels of importance ranging from A–C.^32^ Class A included dietary fat quality (fat in cold and warm meals) and intake of fruit and vegetables. Class B included intake of fish (considered healthy) and sucrose sweetened beverages (considered unhealthy). Class C included cold meat cuts and fast foods (ultra processed foods rich in salt). Based in the food frequency questionnaire individuals were divided into two categories. Non-adherence to dietary guidelines were defined as two class A answers in disagreement with guidelines, and one or two class B answers in disagreement, or three or four class A answers contrasting with current guidelines. Also, a value for dietary adherence on a continuous scale was calculated as 3 times the value of class A answer plus 2 times the value of class B answer plus the value of class C answer.

### Procedures

BMI (kg/m^2^) was calculated using measured weight (kg) divided by measured height squared (m^2^). Routine hospital assays were used to measure non-fasting plasma total cholesterol, triglycerides, apolipoprotein B (apoB), high-density lipoprotein (HDL) cholesterol, and glucose. LDL cholesterol was calculated using the Friedewald equation when triglycerides were less than 4 mmol/L (354 mg/dL) and otherwise measured directly. Remnant cholesterol was calculated as total cholesterol minus LDL cholesterol minus HDL cholesterol and non-HDL cholesterol was calculated as total cholesterol minus HDL cholesterol. Lipoprotein(a) total mass was measured using largely isoform-insensitive turbidimetric apolipoprotein(a) assays.

High-throughput nuclear magnetic resonance (NMR) spectroscopy was used to measure non-fasting cholesterol content of lipoprotein subfractions in plasma. Lipoprotein subfractions were separated according to size and density and included four overall groups: very-low-density lipoprotein (VLDL) subfractions including chylomicrons and extra-extra-large (XXL) VLDL, extra-large (XL) VLDL, large VLDL, medium VLDL, small VLDL, and extra small VLDL; intermediate-density lipoproteins (IDL); LDL subfractions including large LDL, medium LDL, and small LDL; and HDL subfractions including XL HDL, large HDL, medium HDL, and small HDL. To preserve cholesterol content of lipoprotein subfractions during long-term storage, samples were stored at −80° until NMR analysis. The NMR analyses were conducted at the Metabolomics Core Facility at the University of Bristol using the Nightingale health assay.^33^^,^^34^

Systolic blood pressure was measured using an automated blood pressure device. Information on use of lipid-lowering therapy (yes/no), and low education defined as < 3 years of education following mandatory primary school was self-reported. Type-2 diabetes was self-reported diabetes, self-reported use of antidiabetic medication, non-fasting plasma glucose >11 mmol/L (198 mg/dL), and/or a registered diabetes diagnose prior to examination obtained from the national Danish Patient Registry using WHO International Classification of Diseases (ICD) codes (ICD-8250 and ICD-10 E11, E13, and E14).

### Outcomes

We included two primary endpoints in our analyses: myocardial infarction and coronary heart disease. Myocardial infarction was ICD-8 code 410 and ICD-10 codes I21-22. Coronary heart disease was constructed as a composite end-point including first occurrence of either non-fatal myocardial infarction (ICD-8410; ICD-10 I21-22), revascularization of coronary arteries according to the Nordic Medico-Statistical Committee (NOMESCO) Classification of Surgical Procedures comprising coronary artery bypass graft (CABG) (NOMESCO: KFNA–KFNE) and percutaneous coronary intervention (PCI) (NOMESCO: KFNG00-05), or coronary heart disease death (ICD-10: I20-25). Information on myocardial infarction, CABG, PCI, and mortality was collected from January 1977 through December 2018 using the Danish Civil Registration System and linking the CGPS to the national Danish Patient Registry. Information on coronary heart disease death was collected until 31 December 2016 using the national Danish Causes of Death Registry.

### Statistical analysis

We used STATA/SE 13.1 and R version 3.6.1. Multivariable adjusted associations of current smoking, physical activity in leisure time, alcohol intake, and dietary adherence with risk of myocardial infarction and coronary heart disease were estimated using Cox proportional hazards regression with 95% confidence intervals. Entry was date of examination (=left truncation), and age was used as underlying timescale (=age adjusted). Individuals were followed from examination and until date of event, emigration (n = 568), death, or end of follow-up in December 2018, which ever came first. Multivariable adjustment included age (underlying timescale), sex, and educational level. There were no major violations of the proportional hazard's assumption.

Lifestyle-related differences in lipid levels according to current smoking, physical activity, alcohol intake, and adherence to dietary guidelines were estimated as the average between group difference for each lipid measurement using a general linear model adjusted for age, sex, educational level, and body mass index. There were no major violations of the assumptions for the general linear regression model, which include linearity, homogeneity of variance, independence of observations (no clustering or correlation), and normally distributed residuals. Normality of residuals was assessed using residual plots, and if residuals showed non-normality, variables were logarithmically transformed and reassessed. To enable simple comparison across the wide range of lipid biomarkers, changes were reported per one standard deviation change. Proportional Venn diagrams were used to illustrate overlap between the four lifestyle risk factors.

We conducted mediation analyses using the method by VanderWeele,^35^ which builds on natural direct and indirect effects and allows for interaction between the exposure variable and the explanatory factor, to evaluate to what extent the associations of unhealthy lifestyle with increased risk of myocardial infarction and coronary heart disease were explained by causal risk factors for myocardial infarction and coronary heart disease, i.e. elevated remnant cholesterol, elevated LDL cholesterol, elevated systolic blood pressure, and type 2 diabetes. Explained proportion with 95% confidence intervals for each exposure-explanator-outcome relationship was estimated; results were truncated at zero excess risk explained, as negative values are difficult to understand even though they may be calculated mathematically. We used continuous values of remnant cholesterol, LDL cholesterol, and systolic blood pressure. The product of coefficients method for mediation analyses was used in sensitivity analyses.^36^

Restricted cubic splines^37^ were used in Cox proportional hazards regression models to evaluate the association on continuous scales of intermediate variables including remnant cholesterol, low-density lipoprotein cholesterol, and systolic blood pressure, and exposure variables including cumulative smoking (packyears), alcohol consumption (g/week), and dietary adherence with risk of myocardial infarction and coronary heart disease. Number of knots between three and seven was chosen according to the lowest value of the Akaike information criteria.^38^ The distribution of remnant cholesterol was highly skewed; therefore, remnant cholesterol was logarithmically transformed to approximate normal distribution.

In sensitivity analyses, we additionally adjusted each lifestyle factor assessed by the other three lifestyle factors. We did not adjust for lipid-lowering therapy in main analyses because it could be a mediator rather than a confounder as higher lipid levels trigger primary care physicians to prescribe lipid-lowering drugs to a higher extent than the overall cardiovascular risk. Nevertheless, results were similar as those reported in analyses additionally adjusted for lipid-lowering therapy.

### Role of funding source

The study was funded by the Independent Research Fund, Denmark [grant no:9039-00360B to BGN], and by Johan Boserup and Lise Boserups Grant [grant no: 20795-24 to MØJ]. George Davey Smith and NMR processing costs were supported by the Medical Research Council Integrative Epidemiology Unit at the University of Bristol MC_UU_00032. Funders had no role in study design, data collection, data analysis, data interpretation, writing of the report, or in the decision to submit the paper for publication.

## Results

Baseline characteristics of the 104,867 individuals combined (58,286 women and 46,581 men) or stratified by current smoking (current versus never/former), physical activity in leisure time (high versus low), alcohol intake (low versus high), and dietary adherence (high versus low) are shown in Table 1. During a median follow-up of 9.2 years, 2484 individuals developed myocardial infarction and 3570 developed coronary heart disease. Of the 104,867 individuals, 17,834 (17.0%) reported current smoking, 50,096 (47.8%) low physical activity, 39,628 (37.8%) high alcohol intake, and 17,387 (16.6%) reported low adherence to dietary guidelines. Baseline characteristics of women and men separately are shown in Table S1 and Table S2. Baseline characteristics of 28,392 individuals (14,772 women and 13,620 men) with available NMR estimated lipid profile are shown in Table S3. Overlap between the four lifestyle risk factors are shown in proportional Venn diagrams in the appendix (Figure S1 and Figure S2).

**Table 1:** Baseline characteristics of all individuals and stratified by smoking status (never/former versus never), physical activity in leisure time (high versus low), alcohol intake (low versus high), and dietary adherence (high versus low).

	All	Smoking status		Physical activity		Alcohol intake		Dietary adherence	
		Never/former	Current	High	Low	Low	High	High	Low
	n = 104,867	n = 87,033	n = 17,834	n = 53,958	n = 50,096	n = 60,654	n = 39,628	n = 78,710	n = 17,387
**Potential confounders**									
Age, y	57.74 (47.97–67.04)	58.06 (47.98–67.48)	56.39 (47.88–64.88)	56.84 (47.25–66.61)	58.36 (48.72–67.50)	55.08 (45.99–65.63)	60.97 (52.25–68.52)	57.52 (48.04–66.69)	57.11 (46.76–67.58)
Women	58,286 (55.6)	48,892 (56.2)	9394 (52.7)	27,666 (51.3)	30,090 (60.1)	39,512 (65.1)	15,426 (38.9)	46,349 (58.9)	6733 (38.7)
Cumulative smoking, pack years	2.40 (0.00–18.00)	0.00 (0.00–11.00)	25.00 (14.00–39.00)	1.20 (0.00–15.00)	4.00 (0.00–22.00)	0.30 (0.00–14.00)	7.00 (0.00–24.29)	1.25 (0.00–15.00)	10.71 (0.00–30.75)
Low educational level	56,461 (53.8)	44,274 (50.9)	12,187 (68.3)	25,819 (47.9)	30,061 (60.0)	33,161 (54.7)	20,077 (50.7)	38,600 (49.0)	12,011 (69.1)
Lipid-lowering therapy	10,717 (10.2)	9047 (10.4)	1670 (9.4)	4853 (9.0)	5730 (11.4)	5453 (9.0)	4851 (12.2)	8006 (10.2)	1512 (8.7)
**Lipid measurements**									
Total cholesterol, mmol/L	5.60 (4.90–6.30)	5.50 (4.90–6.30)	5.70 (5.00–6.40)	5.50 (4.80–6.20)	5.60 (4.90–6.40)	5.50 (4.80–6.20)	5.70 (5.00–6.40)	5.50 (4.90–6.30)	5.60 (4.90–6.40)
Total cholesterol, mg/dL	216.55 (189.48–243.62)	212.68 (189.48–243.62)	220.42 (193.35–247.49)	212.68 (185.62–239.75)	216.55 (189.48–247.49)	212.68 (185.62–239.75)	220.42 (193.35–247.49)	212.68 (189.48–243.62)	216.55 (189.48–247.49)
Non-HDL cholesterol, mmol/L	3.90 (3.20–4.68)	3.86 (3.17–4.63)	4.11 (3.37–4.93)	3.81 (3.13–4.58)	4.00 (3.29–4.79)	3.85 (3.16–4.63)	3.98 (3.27–4.75)	3.86 (3.17–4.63)	4.08 (3.36–4.88)
Non-HDL cholesterol, mg/dL	150.81 (123.74–180.98)	149.27 (122.58–179.04)	158.93 (130.32–190.64)	147.33 (121.04–177.11)	154.68 (127.22–185.23)	148.88 (122.20–179.04)	153.91 (126.45–183.68)	149.27 (122.58–179.04)	157.77 (129.93–188.71)
Remnant cholesterol, mmol/L	0.62 (0.43–0.92)	0.60 (0.42–0.89)	0.71 (0.49–1.04)	0.58 (0.41–0.86)	0.66 (0.46–0.98)	0.60 (0.42–0.89)	0.65 (0.45–0.96)	0.60 (0.42–0.88)	0.72 (0.49–1.06)
Remnant cholesterol, mg/dL	23.98 (16.63–35.58)	23.20 (16.24–34.42)	27.46 (18.95–40.22)	22.43 (15.85–33.26)	25.72 (17.79–37.90)	23.20 (16.24–34.22)	25.14 (17.40–37.12)	23.20 (16.24–34.03)	27.84 (18.95–40.99)
LDL cholesterol, mmol/L	3.20 (2.60–3.85)	3.20 (2.60–3.80)	3.30 (2.70–4.00)	3.15 (2.59–3.80)	3.27 (2.60–3.90)	3.20 (2.60–3.80)	3.24 (2.60–3.90)	3.20 (2.60–3.80)	3.30 (2.70–3.92)
LDL cholesterol, mg/dL	123.74 (100.54–148.88)	123.74 (100.54–146.95)	127.61 (104.41–154.68)	121.81 (100.16–146.95)	126.45 (100.54–150.81)	123.74 (100.54–146.95)	125.29 (100.54–150.81)	123.74 (100.54–146.95)	127.61 (104.41–151.59)
HDL cholesterol, mmol/L	1.56 (1.25–1.94)	1.59 (1.27–1.96)	1.45 (1.15–1.82)	1.60 (1.29–1.98)	1.52 (1.21–1.90)	1.53 (1.22–1.90)	1.63 (1.30–2.03)	1.59 (1.27–1.97)	1.44 (1.15–1.81)
HDL cholesterol, mg/dL	60.33 (48.34–75.02)	61.49 (49.11–75.79)	56.07 (44.47–70.38)	61.87 (49.88–76.57)	58.78 (46.79–73.47)	59.17 (47.18–73.47)	63.03 (50.27–78.50)	61.49 (49.11–76.18)	55.68 (44.47–69.99)
Lipoprotein (a), mg/dL	9.64 (4.70–28.65)	9.70 (4.74–28.90)	9.36 (4.53–27.61)	9.60 (4.76–28.11)	9.71 (4.65–29.39)	9.77 (4.81–28.85)	9.42 (4.58–28.42)	9.82 (4.83–29.20)	8.91 (4.34–25.85)
Lipoprotein(a), nmol/L	17.19 (6.42–58.63)	17.32 (6.50–59.18)	16.58 (6.04–56.35)	17.09 (6.55–57.45)	17.34 (6.32–60.25)	17.47 (6.66–59.06)	16.70 (6.15–58.13)	17.57 (6.70–59.82)	15.60 (5.63–52.51)
**Within biological pathway**									
Systolic blood pressure, mmHg	140.00 (126.00–155.00)	140.00 (127.00–155.00)	138.00 (125.00–152.00)	139.00 (125.00–154.00)	140.00 (127.00–156.00)	137.00 (124.00–152.00)	143.00 (130.00–158.00)	140.00 (126.00–154.00)	140.00 (128.00–155.00)
C-reactive protein, mg/L	1.39 (0.93–2.26)	1.35 (0.90–2.13)	1.64 (1.09–3.00)	1.29 (0.85–1.94)	1.53 (1.03–2.67)	1.36 (0.90–2.22)	1.41 (0.96–2.23)	1.34 (0.90–2.11)	1.58 (1.05–2.85)
BMI, kg/m^2^	25.55 (23.22–28.40)	25.60 (23.26–28.44)	25.34 (22.98–28.19)	25.08 (22.96–27.60)	26.15 (23.55–29.27)	25.37 (22.98–28.36)	25.74 (23.56–28.31)	25.40 (23.13–28.18)	26.06 (23.53–28.99)
Diabetes mellitus	4984 (4.8)	4144 (4.8)	840 (4.7)	2072 (3.8)	2845 (5.7)	2810 (4.6)	1886 (4.8)	3673 (4.7)	758 (4.4)

Values are median (interquartile range) for continuous variables and number of individuals (%) for categorial variables. Baseline characteristics are based on information from the date of examination. Number of individuals vary slightly for individual covariates dependent on availability of covariate. Abbreviations: BMI = body mass index; HDL = high-density lipoprotein; LDL = low-density lipoproteins.

### Unhealthy lifestyle risk factors and myocardial infarction and coronary heart disease

**Fig. 2:**
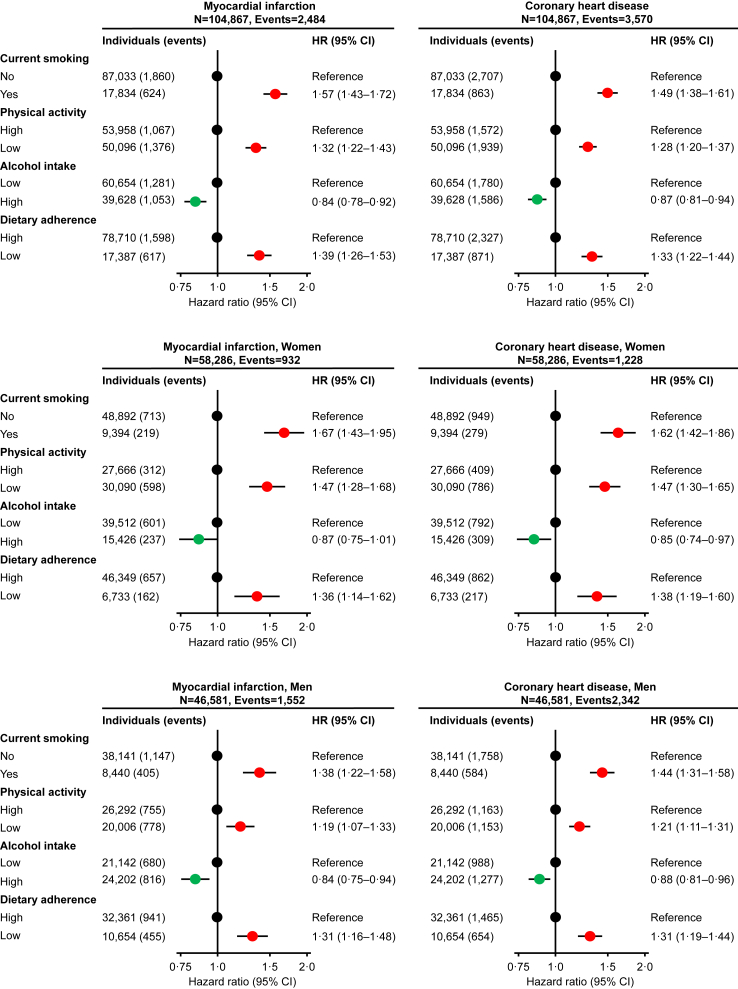
**Risk of myocardial infarction and coronary heart disease according to smoking status–physical activity–alcohol intake–and dietary adherence**. A total of 104,867 individuals from the Copenhagen General Population Study were included in the analyses. During a median follow-up of 9.2 years (ranging from 0 to 15 years), 2484 individuals developed myocardial infarction and 3570 coronary heart disease. Hazard ratios were multivariable adjusted for age (underlying timescale)–sex–educational level–and body mass index. HR = hazard ratio. CI = confidence interval.

In multivariable adjusted analyses, current smoking versus never/former smoking had hazard ratios of 1.57 (95% confidence interval (CI): 1.43–1.72) for myocardial infarction and 1.49 (1.38–1.61) for coronary heart disease. Corresponding hazard ratios for low versus high physical activity were 1.32 (1.22–1.43) and 1.28 (1.20–1.37), for high versus low alcohol intake 0.84 (0.78–0.92) and 0.87 (0.81–0.94), and for low versus high dietary adherence 1.39 (1.26–1.53) and 1.33 (1.22–1.44), respectively (Fig. 2, upper panel). Results were similar in women (Fig. 2, middle panel) and in men (Fig. 2, bottom panel). The shapes of the association of cumulative smoking, alcohol intake, and dietary adherence on continuous scale with myocardial infarction and coronary heart disease are shown in Figure S3. Results were similar when each lifestyle factor were additionally adjusted for the other three lifestyle factors (compare Figure S4 with Fig. 2).

### Combination of unhealthy lifestyle risk factors and myocardial infarction and coronary heart disease

**Fig. 3:**
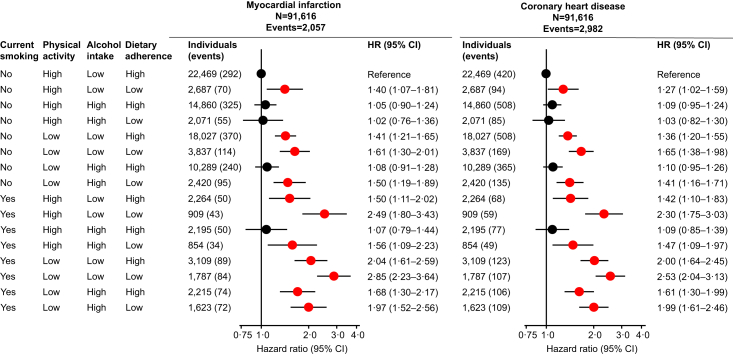
**Risk of myocardial infarction and coronary heart disease according to four factor combinations of lifestyle factors**. A total of 91,616 individuals from the Copenhagen General Population Study with complete information on smoking status, physical activity, alcohol intake, and dietary adherence were included in these analyses. During a median follow-up of 9.0 years (ranging from 0 to 15 years), 2057 individuals developed myocardial infarction and 2982 developed coronary heart disease. Hazard ratios were multivariable adjusted for age (underlying timescale), sex, and educational level. HR = hazard ratio. CI = confidence interval.

**Fig. 4:**
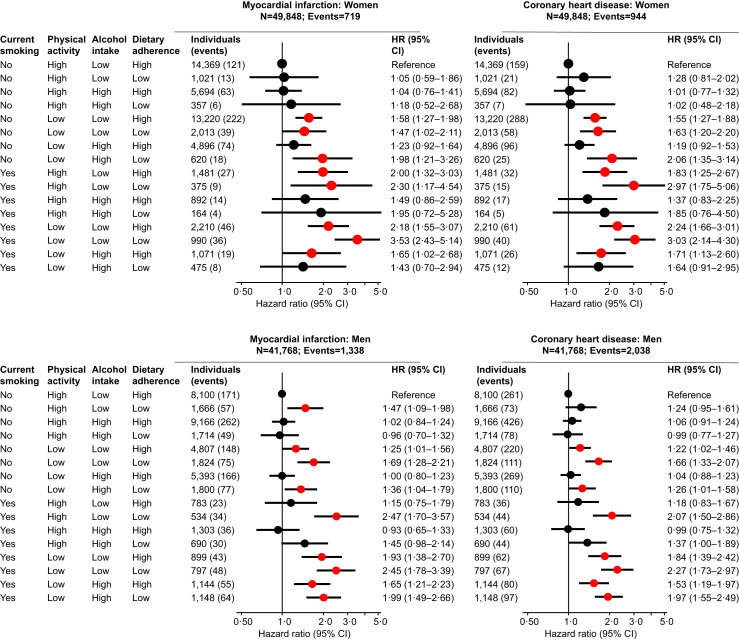
**Risk of myocardial infarction and coronary heart disease according to four factor combinations of lifestyle factors in women and men, separately**. A total of 49,848 women and 41,768 men from the Copenhagen General Population Study with complete information on smoking status, physical activity, alcohol intake, and dietary adherence were included in these analyses. During a median follow-up of 9.0 years (ranging from 0 to 15 years), 719 women and 1338 men developed myocardial infarction and 944 women and 2038 men developed coronary heart disease. Hazard ratios were multivariable adjusted for age (underlying timescale), sex, and educational level. HR = hazard ratio. CI = confidence interval.

When compared to individuals with smoking status never or former, high physical activity, low alcohol intake, and high dietary adherence, individuals with smoking status current, low physical activity, low alcohol intake, and low dietary adherence had the highest risk of myocardial infarction and coronary heart disease with hazard ratios of 2.85 (2.23–3.64) for myocardial infarction and 2.53 (2.04–3.13) for coronary heart disease (Fig. 3). Results were largely similar in women and men, with corresponding hazard ratios of 3.53 (2.43–5.14) for myocardial infarction and 3.03 (2.14–4.30) for coronary heart disease in women (Fig. 4, upper panel), and 2.45 (1.78–3.39) for myocardial infarction and 2.27 (1.73–2.97) for coronary heart disease in men (Fig. 4, lower panel). Results were similar if we added residuals of lifestyle factors to the multivariable adjustment, and if we excluded individuals with diagnosed heart failure, ischaemic heart disease, chronic obstructive pulmonary disease, ischaemic stroke, or non-skin cancer prior to examination.

### Elevated remnant cholesterol from unhealthy lifestyle

**Fig. 5:**
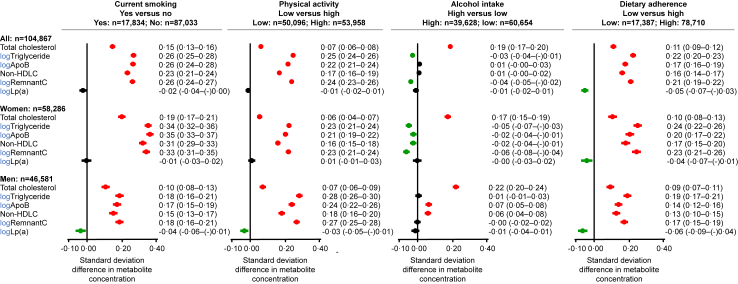
**Standard lipid measurements according to unhealthy lifestyle**. A total of 104,867 individuals from the Copenhagen General Population Study were included in these analyses. Skewed variables were logarithmically (log) transformed. Standard deviation differences between groups are shown with 95% CI. Estimates were adjusted for age; sex, and educational level. ApoB = apolipoprotein B. HDL-C = high-density lipoprotein cholesterol. Lp(a) = lipoprotein(a).

In analyses of lifestyle-related differences in lipid levels, current smoking, low physical activity, and low dietary adherence were each associated with higher cholesterol in very low-density lipoproteins, that is, higher remnant cholesterol, with corresponding higher plasma triglycerides, non-HDL cholesterol, and apolipoprotein B (Fig. 5). In contrast, high alcohol intake was associated with slightly lower remnant cholesterol.

**Fig. 6:**
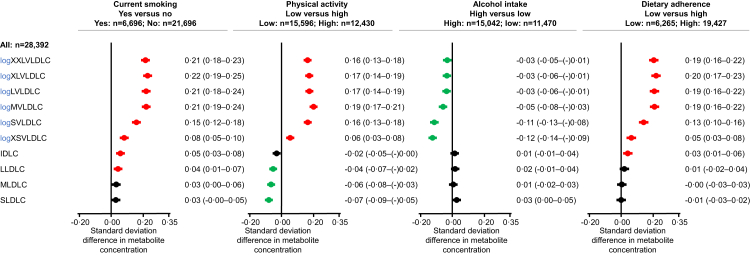
**Nuclear magnetic resonance (NMR) lipid measurements according to unhealthy lifestyle**. A total of 28,392 individuals nested within the Copenhagen General Population Study with NMR measurements available were included in the analyses. Lipid subfractions were estimated by NMR spectroscopy. Skewed variables were logarithmically (log) transformed. Standard deviation differences between groups are shown with 95% CI. Estimates were adjusted for age, sex, and educational level. VLDL = Very low-density lipoproteins. IDL = intermediate-density lipoproteins. LDL = low-density lipoproteins. XXL = extra extra large. XL = extra large. L = large. M = medium. S = small. XS = extra small. NMR = nuclear magnetic resonance. CI = confidence interval.

Similarly, in analyses of lifestyle-related differences in cholesterol content of subfractions of very low-density lipoproteins, intermediate-density lipoproteins, and low-density lipoproteins, current smoking, low physical activity, and low dietary adherence were each associated with higher cholesterol in very low-density lipoproteins, and high alcohol intake was associated with slightly lower cholesterol in very low-density lipoproteins (Fig. 6). Results were similar in women and men, although more pronounced in women than in men (Figure S5). Results were similar to those reported if we excluded individuals with diagnosed heart failure, ischaemic heart disease, chronic obstructive pulmonary disease, ischaemic stroke, or non-skin cancer prior to examination.

### Excess risk explained by elevated remnant cholesterol

**Fig. 7:**
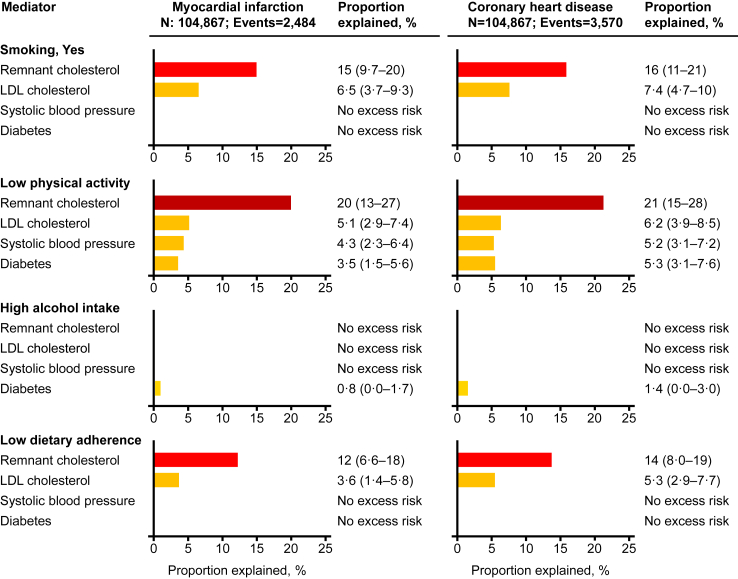
**Explained excess risk from unhealthy lifestyle through cardiovascular risk factors**. Percent excess risk of myocardial infarction and coronary heart disease from unhealthy lifestyle explained by intermediate variables in 104,867 individuals from the Copenhagen General Population Study using the method by VanderWeele.^35^ During a median follow-up of 9.2 years (ranging from 0 to 15 years), 2484 individuals developed myocardial infarction and 3570 developed coronary heart disease. Estimated excess risk in percent with 95% CI for each exposure-mediator-outcome relationship are shown. Estimates were multivariable adjusted for age (underlying time scale), sex, and educational level, and truncated at zero excess risk explained. LDL = low-density lipoprotein.

**Fig. 8:**
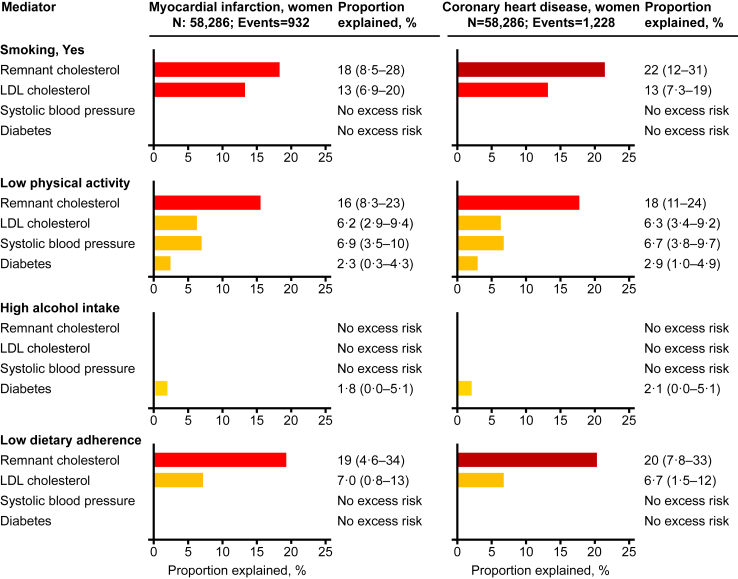
**Explained excess risk from unhealthy lifestyle through cardiovascular risk factors in women**. Percent excess risk of myocardial infarction and coronary heart disease from unhealthy lifestyle explained by intermediate variables in 58,286 women from the Copenhagen General Population Study using the method by VanderWeele.^35^ During a median follow-up of 9.2 years (ranging from 0 to 15 years), 932 women developed myocardial infarction and 1228 developed coronary heart disease. Estimated excess risk in percent with 95% CI for each exposure-mediator-outcome relationship are shown. Estimates were multivariable adjusted for age (underlying time scale), sex, and educational level, and truncated at zero excess risk explained. LDL = low-density lipoprotein.

**Fig. 9:**
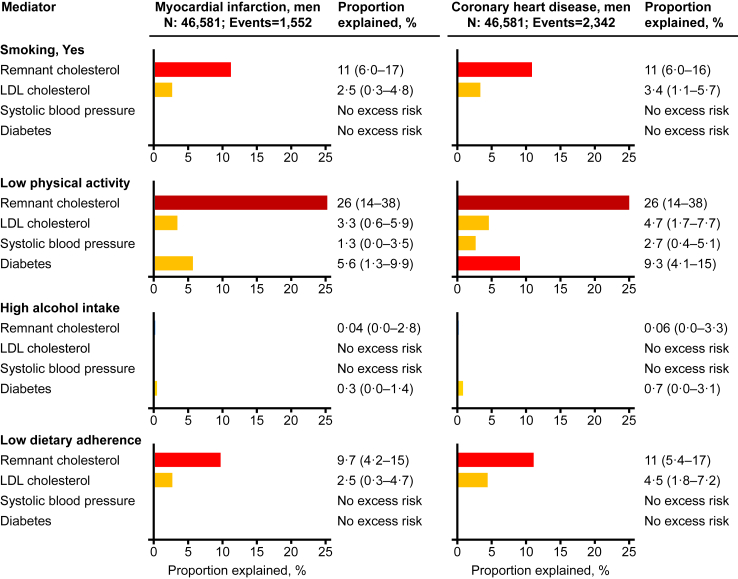
**Explained excess risk from unhealthy lifestyle through cardiovascular risk factors in men**. Percent excess risk of myocardial infarction and coronary heart disease from unhealthy lifestyle explained by intermediate variables in 46,581 men from the Copenhagen General Population Study using the method by VanderWeele.^35^ During a median follow-up of 9.2 years (ranging from 0 to 15 years), 1552 men developed myocardial infarction and 2342 developed coronary heart disease. Estimated excess risk in percent with 95% CI for each exposure-mediator-outcome relationship are shown. Estimates were multivariable adjusted for age (underlying time scale), sex, and educational level, and truncated at zero excess risk explained. LDL = low-density lipoprotein.

In mediation analyses, we observed that for current smoking, remnant cholesterol explained 15% (95% confidence interval: 9.7%–20%) of the excess risk of myocardial infarction and 16% (11%–21%) of the excess risk of coronary heart disease (Fig. 7). Corresponding values for low physical activity were 20% (13%–27%) and 21% (15%–28%), and for low adherence to dietary guidelines 12% (6.6%–18%) and 14% (8.0%–19%), respectively. Results were similar in women (Fig. 8) and in men (Fig. 9). Also, results were similar when each lifestyle factor were additionally adjusted for the other three lifestyle factors (compare Figure S6 with Fig. 7), when using the product of coefficients methods (compare Figure S7 with Fig. 7), and when using different definitions of smoking status (compare Figure S8 with Fig. 7, upper panel). The shapes of the association of remnant cholesterol, LDL cholesterol, and systolic blood pressure with myocardial infarction and coronary heart disease are shown in Figure S9. Individuals with versus without diabetes had hazard ratios of 1.50 (1.31%–1.71%) for myocardial infarction and 1.61 (1.45%–1.80%) for coronary heart disease. In individuals with diabetes only (N = 4984), with much lower statistical power than in the overall analyses, we did not observe statistically significant excess risk explained by remnant cholesterol for any of the four unhealthy lifestyle factors (Figure S10).

## Discussion

In this prospective cohort study of 104,867 individuals (58,286 women and 46,581 men) from the Copenhagen General Population Study, we found that elevated remnant cholesterol explained a substantial proportion of the increased risk of myocardial infarction and coronary heart disease in individuals with unhealthy lifestyle. Clinically, these novel findings underline the importance of both elevated remnant cholesterol and promotion of healthy lifestyle in primary prevention of myocardial infarction and coronary heart disease.

Our findings that higher remnant cholesterol explains a substantial proportion of increased risk of myocardial infarction and coronary heart disease in individuals with unhealthy lifestyle seems biological plausible. Higher remnant cholesterol in the blood reflects a higher concentration of VLDL particles^39^ that may penetrate the arterial intima and be more easily trapped in the arterial intima than LDL particles due to their larger size.^12^^,^^40^ Once trapped in the arterial wall, they cause local inflammation^12^ and foam cell formation^41^ leading to formation of atherosclerotic plaques, which may lead to coronary heart disease.^12^^,^^42^^,^^43^ Likewise, the association of smoking, physical inactivity, and unhealthy diet with higher remnant cholesterol also seems biological plausible. First, higher remnant cholesterol with smoking is likely explained by smoking-induced decrease in lipoprotein lipase (LPL) activity and higher catecholamine level; the latter resulting in insulin resistance and higher plasma glucose, and activated lipolysis with increased release of free fatty acids, both of which are substrates for triglyceride synthesis and thus triglyceride-rich VLDL particle formation.^44^ Second, higher remnant cholesterol with physical inactivity is likely explained by low energy demand and thus low level of triglyceride metabolism by muscle cells leading to accumulation of triglyceride-rich VLDL particles carrying remnant cholesterol.^45^ Third, higher remnant cholesterol with unhealthy diet is likely attributable to increased intake of saturated fats and fast food in contrast to unsaturated fatty acids from fish, vegetables, and fruits. Higher saturated fatty acids and triglycerides subsequently leads to an increase in formation of remnant cholesterol contained in VLDL particles.

In this study, alcohol intake was associated with a lower risk of myocardial infarction and coronary heart disease, which may seem counterintuitive. Nevertheless, this association has been shown in several studies.^46^ Potential explanations include the association between alcohol and higher levels of high-density lipoprotein (HDL) cholesterol as well as the anticoagulant effects of alcohol.^47^ Additionally, moderate alcohol consumption is often linked to other healthy lifestyle factors, such as regular physical activity and a nutritious diet, which together contribute to improved cardiovascular health.^48^ Therefore, while moderate alcohol intake is associated with reduced cardiovascular risk, this relationship is complex and unlikely to reflect a straightforward cause-and-effect relationship.

In support of our cohort as a valid population sample for the present study question, observational associations between unhealthy lifestyle and increased risk of coronary heart disease have also been found in several studies studies.^2^^,^^10^^,^^49^, ^50^, ^51^, ^52^, ^53^, ^54^ Further, studies have reported higher VLDL and lower HDL with smoking,^25^^,^^26^ low physical activity,^24^^,^^25^ and unhealthy diet.^25^

In support of our findings that remnant cholesterol explains a substantial proportion of increased risk of coronary heart disease in unhealthy lifestyle, Ahmad et al.^55^ observed that lower remnant cholesterol explained 21% of reduced risk of cardiovascular disease associated with a Mediterranean diet in 25,317 individuals from the Women's Health Study. Further, Lu et al.^56^ reported that VLDL explained part of the association between smoking and unhealthy diet with risk of cardiovascular disease in 5072 individuals with diabetes from the UK Biobank. Also, Si et al.^57^ found that lower VLDL was associated with adherence to a combined healthy lifestyle explained 5–10% of reduced risk of coronary heart disease in 4681 individuals from the prospective China Kadoorie Biobank. However, the fraction of excess risk of myocardial infarction and coronary heart disease explained by higher remnant cholesterol with smoking, low physical activity, or unhealthy diet in a large general population sample have not been investigated before.

Ideally elevated remnant cholesterol due to unhealthy lifestyle should be corrected by improvement of lifestyle including recommendations to stop smoking, increase physical activity, and to increase intake of fruit and vegetables, to use unsaturated fat instead of saturated fat for warm and cold meals, to increase intake of fish, and to reduce intake of sugar, refined carbohydrates, and fast food.^58^^,^^59^ Further, individuals with overweight and obesity are recommended to lose weight.^58^^,^^59^ However, many individuals struggle to change lifestyle, why pharmaceutical therapy for elevated remnant cholesterol may be relevant for a number of individuals including pharmacological treatment with LDL cholesterol-lowering statins, PCSK9-inhibitors, ezetimibe, and triglyceride- and remnant cholesterol-lowering fibrates and high-dose fish oil.^8^^,^^59^ Furthermore, as obesity is an important driver of elevated remnant (VLDL) cholesterol, pharmacologic treatment of obesity with glucagon-like peptide-1 (GLP-1) analogues likewise have the potential to reduce remnant cholesterol. In support, treatment with semaglutide has been associated a relative reduction in VLDL cholesterol of 22%.^60^ Finally, novel therapeutic agents are under development aiming at reducing risk of ASCVD by reducing plasma triglycerides, apoB-containing lipoproteins, and remnant cholesterol and include agents that inhibit apolipoprotein C3 (ApoC3) and angiopoietin-related protein-3 (ANGPTL3); both of which efficiently lowers plasma triglycerides.^61^^,^^62^ For example, small interfering RNAs designed to reduce hepatic synthesis and secretion of ApoC3 and ANGTPL3 given to individuals with mixed hyperlipidemia, reduced remnant cholesterol up to 49 and 82 percentage points at week 24 as compared with placebo.^61^^,^^62^

Strengths of our study include a large sample size with long follow-up time, high accuracy of diagnostic information through nation-wide Danish health registers,^63^ no loss to follow-up, the use of standardised and validated lipid measurements, and that we were able to include directly measured subfractions of lipid measurements using NMR spectroscopy.

Limitations of the study include that a formal mediation analysis require causality between exposure, mediator, and outcome, which has not been assessed for all variables; however, it is widely accepted that unhealthy lifestyle influences blood cholesterol,^58^^,^^59^ blood pressure,^64^^,^^65^ diabetes risk,^66^^,^^67^ and cardiovascular risk.^8^^,^^9^ Also, the fact that our dietary assessment was based on a simple and short food frequency questionnaire could be considered a limitation; however, the manageable and simple construction of the food frequency questionnaire resulted in a high response rate. Further, a previous study using a similar short food frequency questionnaire found that the assessed dietary quality was comparable to a more extensive food frequency questionnaire.^68^ Another limitation is that the measures that correlate with the development of the long-term disease were at a specific time. In addition, Denmark is a high-income, low-risk country with universal health care access, which limits generalizability; however, we are not aware of data to suggest that the present result should not apply to people living in many other countries. Finally, we only included white adults of Danish descent. Therefore, our result may not necessarily apply to other ethnicities; however, we are not aware of any data suggesting that our results should not apply to all ethnicities. That said, genetics and lifestyle vary across ethnicities and regions, meaning that the impact of elevated remnant cholesterol from an unhealthy lifestyle may depend on what is typically considered unhealthy in each specific population.

When evaluating and interpreting observational epidemiological research, it is important to consider potential selection bias, as those who accept an invitation to participate may not fully represent the general population. In the Copenhagen General Population Study, the participation rate was 43%, which is relatively high compared to other large population-based prospective cohort studies. However, it is well-known that responders and non-responders often differ in lifestyle and health. Generally, non-responders are more likely to have an unfavourable lifestyle, and comorbidities are often more prevalent among them, whereas those who participate tend to be at lower risk, suggesting that the observed results may be conservative.

In conclusion, the present data suggest that elevated remnant cholesterol explains part of the excess myocardial infarction and coronary heart disease events in individuals with unhealthy lifestyle. Clinically, these novel findings support focus on promoting a healthy lifestyle throughout life and support focus on elevated remnant cholesterol for prevention of myocardial infarction and coronary heart disease.

## Contributors

All authors participated in the study design, generation of the aims of the study, and interpretation of the data. MØJ, GDS, and BGN acquired funding. MØJ conducted statistical analyses. SA independently verified the results reported in the manuscript. MØJ wrote the original draft of the report. SA, SVK, SFN, GSD, and BGN critically reviewed and edited the original draft of the report.

## Data sharing statement

The Danish Data Protection Agency does not allow open access to our data; however, upon reasonable request, the corresponding author can allow joint analyses with other researchers.

## Declaration of interests

BGN report consultancies and talks sponsored by AstraZeneca, Sanofi, Regeneron, Ionis, Amgen, Kowa, Denka, Amarin, Novartis, Novo Nordisk, Esperion, Abbott, Silence Therapeutics, Ultragenyx, USV, Mankind, Lilly, Arrowhead, and Marea, and President-Elect of European Atherosclerosis Society. SA report support from Abbott for attending a meeting. GDS reports Scientific Advisory Board Membership for Relation Therapeutics and Insitro. There are no financial or other conflicts of interest for MØJ, SVK, or SFN.
