# Supplementary material for: Association of remnant cholesterol with unhealthy lifestyle and risk of coronary heart disease: a population-based cohort study
Source: Lancet Reg Health Eur. 2025 Feb 7;51:101223. doi: 10.1016/j.lanepe.2025.101223 (PMC11984604; doi:10.1016/j.lanepe.2025.101223)
Supplement: Supplementary Figures and Tables [file mmc1.pdf]

Supplementary Appendix

This appendix has been provided by the authors to give readers additional information about their work.

Supplement to:

Johansen MO, Vedel-Krogh S, Nielsen SF, et al. Elevated remnant cholesterol and excess coronary heart disease in unhealthy lifestyle: a population-based study of women and men

Table of contents:

Table S1: Baseline characteristics stratified by smoking status (never/former versus never), physical activity in leisure time (high versus low), alcohol intake (low versus high), and dietary adherence (high versus low) in 58,286 women. 2

Table S2: Baseline characteristics stratified by smoking status (never/former versus never), physical activity in leisure time (high versus low), alcohol intake (low versus high), and dietary adherence (high versus low) in 46,581 men. 5

Table S3: Baseline characteristics stratified by smoking status (never/former versus never), physical activity in leisure time (high versus low), alcohol intake (low versus high), and dietary adherence (high versus low) in 28,392 individuals with available NMR estimated lipid profile. 8

Figure S1: Proportional Venn Diagram showing percent of individuals with each lifestyle factor 11

Figure S2: Proportional Venn Diagram showing number of individuals with each lifestyle factor. 12

Figure S3: Risk of myocardial infarction, coronary heart disease, and all-cause mortality by exposure variables on continuous scale. 13

Figure S4: Risk of myocardial infarction and coronary heart disease according to smoking status, physical activity, alcohol intake, and dietary adherence with each lifestyle factor additionally adjusted for the three other lifestyle factors. 14

Figure S5: NMR lipid measurements according to unhealthy lifestyle in women and men, separately. 15

Figure S6: Explained excess risk from unhealthy lifestyle with each lifestyle factor additionally adjusted for the other three lifestyle factors 16

Figure S7: Explained excess risk in unhealthy lifestyle using the product of coefficients method 17

Figure S8: Explained excess risk in unhealthy lifestyle using different definitions of smoking status 18

Figure S9: Risk of myocardial infarction, coronary heart disease, and all-cause mortality by intermediate variables on continuous scale. 20

Figure S10: Explained excess risk in unhealthy lifestyle in individuals with type 2 diabetes 21

References 22

Table S1: Baseline characteristics stratified by smoking status (never/former versus never), physical activity in leisure time (high versus low), alcohol intake (low versus high), and dietary adherence (high versus low) in 58,286 women.

	All	Smoking status		Physical activity	
		Never/former	Current	High	Low
	n=58,286	n=48,892	n=9,394	n=27,666	n=30,090
**Potential confounders**					
Age, y	57·53 (47·69–66·80)	58·01 (47·79–67·27)	55·39 (47·29–63·94)	58·01 (47·79–67·27)	55·39 (47·29–63·94)
Women	58286 (100·0)	48892 (100·0)	9394 (100·0)	48892 (100·0)	9394 (100·0)
Cumulative smoking, pack years	1·00 (0·00–14·00)	0·00 (0·00–7·50)	22·50 (12·00–34·00)	0·00 (0·00–7·50)	22·50 (12·00–34·00)
Low educational level	33273 (57·1)	26539 (54·3)	6734 (71·7)	26539 (54·3)	6734 (71·7)
Lipid-lowering therapy	5485 (9·4)	4657 (9·5)	828 (8·8)	4657 (9·5)	828 (8·8)
**Lipid measurements**					
Total cholesterol, mmol/L	5·60 (4·90–6·40)	5·60 (4·90–6·30)	5·70 (5·00–6·50)	5·60 (4·90–6·30)	5·70 (5·00–6·50)
Total cholesterol, mg/dL	216·55 (189·48–247·49)	216·55 (189·48–243·62)	220·42 (193·35–251·35)	216·55 (189·48–243·62)	220·42 (193·35–251·35)
Non-HDL cholesterol, mmol/L	3·78 (3·10–4·55)	3·74 (3·06–4·49)	4·03 (3·32–4·85)	3·74 (3·06–4·49)	4·03 (3·32–4·85)
Non-HDL cholesterol, mg/dL	146·17 (119·88–175·95)	144·63 (118·33–173·63)	155·84 (128·38–187·55)	144·63 (118·33–173·63)	155·84 (128·38–187·55)
Remnant cholesterol, mmol/L	0·55 (0·39–0·79)	0·53 (0·39–0·77)	0·64 (0·45–0·93)	0·53 (0·39–0·77)	0·64 (0·45–0·93)
Remnant cholesterol, mg/dL	21·27 (15·27–30·55)	20·50 (15·08–29·78)	24·75 (17·40–35·96)	20·50 (15·08–29·78)	24·75 (17·40–35·96)
LDL cholesterol, mmol/L	3·20 (2·60–3·80)	3·13 (2·55–3·80)	3·30 (2·70–4·00)	3·13 (2·55–3·80)	3·30 (2·70–4·00)
LDL cholesterol, mg/dL	123·74 (100·54–146·95)	121·04 (98·61–146·95)	127·61 (104·41–154·68)	121·04 (98·61–146·95)	127·61 (104·41–154·68)
HDL cholesterol, mmol/L	1·74 (1·42–2·10)	1·76 (1·45–2·12)	1·59 (1·29–1·97)	1·76 (1·45–2·12)	1·59 (1·29–1·97)
HDL cholesterol, mg/dL	67·29 (54·91–81·21)	68·06 (56·07–81·98)	61·49 (49·88–76·18)	68·06 (56·07–81·98)	61·49 (49·88–76·18)
Lipoprotein (a), mg/dL	10·20 (5·00–31·05)	10·24 (5·02–31·23)	9·96 (4·86–30·36)	10·24 (5·02–31·23)	9·96 (4·86–30·36)
Lipoprotein(a), nmol/L	18·40 (7·07–63·87)	18·48 (7·12–64·26)	17·88 (6·76–62·36)	18·48 (7·12–64·26)	17·88 (6·76–62·36)
**Within biological pathway**					
Systolic blood pressure, mmHg	136·00 (123·00–152·00)	137·00 (123·00–153·00)	134·00 (120·00–150·00)	137·00 (123·00–153·00)	134·00 (120·00–150·00)
C-reactive protein, mg/L	1·40 (0·94–2·34)	1·37 (0·92–2·22)	1·62 (1·08–3·00)	1·37 (0·92–2·22)	1·62 (1·08–3·00)
BMI, kg/m^2	24·75 (22·46–27·92)	24·77 (22·49–27·94)	24·61 (22·29–27·81)	24·77 (22·49–27·94)	24·61 (22·29–27·81)
Diabetes mellitus	2329 (4·0)	1953 (4·0)	376 (4·0)	1953 (4·0)	376 (4·0)

Alcohol intake		Dietary adherence	
Low	High	High	Low
n=39,512	n=15,426	n=46,349	n=6,733
55·67 (46·34–65·97)	61·12 (52·88–68·12)	57·16 (47·66–66·34)	58·16 (46·69–68·55)
39512 (100·0)	15426 (100·0)	46349 (100·0)	6733 (100·0)
0·15 (0·00–11·25)	4·00 (0·00–18·00)	0·50 (0·00–11·40)	7·50 (0·00–26·00)
22426 (56·8)	8430 (54·6)	24531 (52·9)	5152 (76·5)
3493 (8·8)	1695 (11·0)	4231 (9·1)	549 (8·2)
5·50 (4·80–6·30)	5·80 (5·20–6·50)	5·60 (4·90–6·30)	5·70 (5·00–6·50)
212·68 (185·62–243·62)	224·29 (201·08–251·35)	216·55 (189·48–243·62)	220·42 (193·35–251·35)
3·76 (3·07–4·52)	3·82 (3·15–4·59)	3·75 (3·07–4·51)	3·96 (3·25–4·75)
145·40 (118·72–174·79)	147·72 (121·81–177·50)	145·01 (118·72–174·40)	153·13 (125·68–183·68)
0·55 (0·39–0·79)	0·55 (0·40–0·79)	0·54 (0·39–0·77)	0·62 (0·44–0·90)
21·27 (15·08–30·55)	21·08 (15·47–30·55)	20·88 (15·08–29·78)	23·98 (17·01–34·80)
3·14 (2·54–3·80)	3·20 (2·60–3·90)	3·15 (2·57–3·80)	3·30 (2·67–3·91)
121·42 (98·22–146·95)	123·74 (100·54–150·81)	121·81 (99·38–146·95)	127·61 (103·25–151·20)
1·69 (1·39–2·03)	1·92 (1·59–2·30)	1·75 (1·44–2·11)	1·66 (1·35–2·04)
65·35 (53·75–78·50)	74·25 (61·49–88·94)	67·67 (55·68–81·59)	64·19 (52·20–78·89)
10·19 (4·99–30·96)	10·18 (5·04–32·30)	10·25 (5·04–31·05)	9·60 (4·71–30·18)
18·38 (7·06–63·66)	18·36 (7·16–66·59)	18·51 (7·16–63·87)	17·10 (6·44–61·97)
135·00 (121·00–151·00)	140·00 (126·00–156·00)	136·00 (122·00–152·00)	137·00 (123·00–154·00)
1·38 (0·92–2·31)	1·40 (0·97–2·21)	1·36 (0·91–2·18)	1·68 (1·09–3·25)
24·77 (22·43–28·05)	24·52 (22·45–27·23)	24·65 (22·43–27·72)	24·97 (22·38–28·59)
1602 (4·1)	543 (3·5)	1791 (3·9)	256 (3·8)

Values are median (interquartile range) for continuous variables and number of individuals (%) for categorial variables. Baseline characteristics are based on information from the date of examination. Number of individuals vary slightly for individual covariates dependent on availability of covariate. Abbreviations: BMI = body mass index; HDL = high-density lipoprotein; LDL = low-density lipoproteins.

Table S2: Baseline characteristics stratified by smoking status (never/former versus never), physical activity in leisure time (high versus low), alcohol intake (low versus high), and dietary adherence (high versus low) in 46,581 men.

	All	Smoking status		Physical activity	
		Never/former	Current	High	Low
	n=46,581	n=38,141	n=8440	n=26,292	n=20,006
**Potential confounders**					
Age, y	58·02 (48·31–67·36)	58·14 (48·22–67·72)	57·50 (48·74–65·68)	57·30 (47·57–67·10)	58·58 (49·32–67·67)
Women	46581 (100·0)	38141 (100·0)	8440 (100·0)	26292 (100·0)	20006 (100·0)
Cumulative smoking, pack years	5·40 (0·00–24·29)	0·50 (0·00–16·43)	30·00 (16·00–45·00)	3·00 (0·00–20·00)	10·00 (0·00–30·00)
Low educational level	23188 (49·8)	17735 (46·5)	5453 (64·6)	11851 (45·1)	11152 (55·7)
Lipid-lowering therapy	5232 (11·2)	4390 (11·5)	842 (10·0)	2635 (10·0)	2546 (12·7)
**Lipid measurements**					
Total cholesterol, mmol/L	5·50 (4·80–6·20)	5·50 (4·80–6·20)	5·60 (4·90–6·30)	5·50 (4·80–6·10)	5·50 (4·90–6·30)
Total cholesterol, mg/dL	212·68 (185·62–239·75)	212·68 (185·62–239·75)	216·55 (189·48–243·62)	212·68 (185·62–235·89)	212·68 (189·48–243·62)
Non-HDL cholesterol, mmol/L	4·06 (3·34–4·82)	4·03 (3·33–4·78)	4·19 (3·44–5·01)	3·97 (3·28–4·72)	4·18 (3·44–4·96)
Non-HDL cholesterol, mg/dL	157·00 (129·16–186·39)	155·84 (128·77–184·84)	162·03 (133·02–193·74)	153·52 (126·84–182·52)	161·64 (133·02–191·80)
Remnant cholesterol, mmol/L	0·73 (0·50–1·07)	0·72 (0·50–1·04)	0·79 (0·54–1·18)	0·68 (0·47–0·99)	0·80 (0·55–1·16)
Remnant cholesterol, mg/dL	28·23 (19·34–41·38)	27·84 (19·33–40·22)	30·55 (20·88–45·63)	26·30 (18·17–38·48)	30·94 (21·27–44·86)
LDL cholesterol, mmol/L	3·22 (2·61–3·90)	3·20 (2·60–3·88)	3·30 (2·69–4·00)	3·20 (2·60–3·80)	3·30 (2·67–3·94)
LDL cholesterol, mg/dL	124·52 (100·93–150·81)	123·74 (100·54–150·04)	127·61 (104·02–154·68)	123·74 (100·54–146·95)	127·61 (103·25–152·36)
HDL cholesterol, mmol/L	1·36 (1·10–1·68)	1·37 (1·11–1·69)	1·30 (1·04–1·62)	1·41 (1·15–1·73)	1·29 (1·04–1·60)
HDL cholesterol, mg/dL	52·59 (42·54–64·97)	52·98 (42·92–65·35)	50·27 (40·22–62·84)	54·52 (44·47–66·90)	49·88 (40·22–61·87)
Lipoprotein (a), mg/dL	9·08 (4·43–25·91)	9·14 (4·46–26·12)	8·80 (4·25–24·88)	9·22 (4·53–25·97)	8·88 (4·27–25·82)
Lipoprotein(a), nmol/L	15·96 (5·83–52·65)	16·10 (5·89–53·12)	15·36 (5·43–50·41)	16·27 (6·04–52·79)	15·53 (5·48–52·46)
**Within biological pathway**					
Systolic blood pressure, mmHg	142·00 (130·00–156·00)	143·00 (130·00–157·00)	140·00 (130·00–155·00)	142·00 (130·00–156·00)	144·00 (130·00–158·00)
C-reactive protein, mg/L	1·37 (0·92–2·18)	1·33 (0·88–2·03)	1·66 (1·10–3·00)	1·29 (0·85–1·94)	1·51 (1·02–2·54)
BMI, kg/m^2	26·33 (24·26–28·80)	26·40 (24·37–28·86)	25·99 (23·78–28·49)	25·92 (24·03–28·14)	26·93 (24·65–29·67)
Diabetes mellitus	2655 (5·7)	2191 (5·7)	464 (5·5)	1204 (4·6)	1425 (7·1)

Alcohol intake		Dietary adherence	
**Low**	**High**	**High**	**Low**
n=21,142	n=24,202	n=32,361	n=10,654
53·96 (45·42–65·01)	60·87 (51·81–68·78)	58·06 (48·57–67·17)	56·60 (46·80–66·88)
21142 (100·0)	24202 (100·0)	32361 (100·0)	10654 (100·0)
1·20 (0·00–18·75)	9·64 (0·00–28·57)	3·50 (0·00–20·00)	13·20 (0·00–35·00)
10735 (50·8)	11647 (48·1)	14069 (43·5)	6859 (64·4)
1960 (9·3)	3156 (13·0)	3775 (11·7)	963 (9·0)
5·40 (4·70–6·10)	5·60 (4·90–6·30)	5·50 (4·80–6·20)	5·60 (4·90–6·30)
208·82 (181·75–235·89)	216·55 (189·48–243·62)	212·68 (185·62–239·75)	216·55 (189·48–243·62)
4·04 (3·33–4·79)	4·08 (3·35–4·85)	4·03 (3·32–4·78)	4·16 (3·44–4·95)
156·23 (128·77–185·23)	157·77 (129·54–187·55)	155·84 (128·38–184·84)	160·87 (133·02–191·42)
0·73 (0·50–1·07)	0·73 (0·50–1·07)	0·71 (0·49–1·03)	0·79 (0·54–1·16)
28·23 (19·33–41·38)	28·04 (19·53–41·38)	27·46 (18·95–39·83)	30·55 (20·88–44·86)
3·20 (2·60–3·86)	3·29 (2·63–3·90)	3·20 (2·60–3·87)	3·30 (2·70–3·92)
123·74 (100·54–149·27)	127·22 (101·70–150·81)	123·74 (100·54–149·65)	127·61 (104·41–151·59)
1·26 (1·03–1·55)	1·46 (1·18–1·78)	1·37 (1·11–1·69)	1·32 (1·07–1·63)
48·72 (39·83–59·94)	56·46 (45·63–68·83)	52·98 (42·92–65·35)	51·04 (41·38–63·03)
9·16 (4·52–25·32)	9·03 (4·33–26·31)	9·28 (4·53–26·55)	8·53 (4·15–24·02)
16·14 (6·03–51·37)	15·85 (5·60–53·53)	16·40 (6·05–54·05)	14·76 (5·21–48·53)
140·00 (129·00–154·00)	145·00 (132·00–160·00)	142·00 (130·00–156·00)	142·00 (130·00–156·00)
1·33 (0·87–2·09)	1·41 (0·95–2·24)	1·32 (0·88–2·02)	1·53 (1·03–2·62)
26·21 (24·11–28·77)	26·41 (24·39–28·78)	26·23 (24·24–28·63)	26·56 (24·27–29·15)
1208 (5·7)	1343 (5·5)	1882 (5·8)	502 (4·7)

Values are median (interquartile range) for continuous variables and number of individuals (%) for categorial variables. Baseline characteristics are based on information from the date of examination. Number of individuals vary slightly for individual covariates dependent on availability of covariate. Abbreviations: BMI = body mass index; HDL = high-density lipoprotein; LDL = low-density lipoproteins.

Table S3: Baseline characteristics stratified by smoking status (never/former versus never), physical activity in leisure time (high versus low), alcohol intake (low versus high), and dietary adherence (high versus low) in 28,392 individuals with available NMR estimated lipid profile.

	All	Smoking status		Physical activity	
		Never/former	Current	High	Low
	n=28,392	n=21,696	n=6,696	n=12,430	n=15,596
**Potential confounders**					
Age, y	62·01 (51·34–72·27)	63·02 (51·77–73·20)	59·34 (50·47–68·52)	61·73 (50·34–70·87)	61·98 (52·01–73·26)
Women	14772 (52·0)	11453 (52·8)	3319 (49·6)	5707 (45·9)	8830 (56·6)
Cumulative smoking, pack years	6·50 (0·00–26·25)	0·50 (0·00–15·18)	30·00 (17·50–43·39)	4·20 (0·00–22·50)	9·00 (0·00–30·00)
Low educational level	18025 (63·5)	13049 (60·1)	4976 (74·3)	7134 (57·4)	10602 (68·0)
Lipid-lowering therapy	3143 (11·1)	2490 (11·5)	653 (9·8)	1205 (9·7)	1881 (12·1)
**Lipid measurements**					
Total cholesterol, mmol/L	5·60 (4·90–6·40)	5·60 (4·90–6·40)	5·70 (5·00–6·50)	5·60 (4·90–6·40)	5·70 (5·00–6·40)
Total cholesterol, mg/dL	216·55 (189·48–247·49)	216·55 (189·48–247·49)	220·42 (193·35–251·35)	216·55 (189·48–247·49)	220·42 (193·35–247·49)
Non-HDL cholesterol, mmol/L	4·00 (3·29–4·79)	3·96 (3·26–4·73)	4·14 (3·40–4·95)	3·94 (3·24–4·73)	4·05 (3·34–4·84)
Non-HDL cholesterol, mg/dL	154·68 (127·22–185·23)	153·13 (126·06–183·01)	160·09 (131·48–191·42)	152·36 (125·29–182·81)	156·61 (129·16–187·16)
Remnant cholesterol, mmol/L	0·67 (0·47–0·99)	0·66 (0·46–0·96)	0·73 (0·50–1·07)	0·64 (0·45–0·94)	0·70 (0·48–1·02)
Remnant cholesterol, mg/dL	25·91 (18·17–38·28)	25·52 (17·79–37·12)	28·23 (19·33–41·38)	24·75 (17·40–36·35)	27·07 (18·56–39·44)
LDL cholesterol, mmol/L	3·23 (2·60–3·90)	3·20 (2·60–3·90)	3·30 (2·70–4·00)	3·20 (2·60–3·90)	3·30 (2·60–3·91)
LDL cholesterol, mg/dL	124·90 (100·54–150·81)	123·74 (100·54–150·81)	127·61 (104·41–154·68)	123·74 (100·54–150·81)	127·61 (100·54–151·20)
HDL cholesterol, mmol/L	1·55 (1·23–1·93)	1·58 (1·25–1·95)	1·47 (1·17–1·84)	1·59 (1·27–1·95)	1·52 (1·20–1·91)
HDL cholesterol, mg/dL	59·94 (47·56–74·63)	61·10 (48·34–75·41)	56·84 (45·24–71·15)	61·49 (49·11–75·41)	58·78 (46·40–73·86)
Lipoprotein (a), mg/dL	9·85 (4·81–29·21)	9·93 (4·84–30·03)	9·64 (4·65–27·08)	9·97 (4·85–30·13)	9·75 (4·79–28·59)
Lipoprotein(a), nmol/L	17·64 (6·66–59·85)	17·81 (6·71–61·63)	17·18 (6·31–55·20)	17·90 (6·73–61·86)	17·41 (6·62–58·50)
**Within biological pathway**					
Systolic blood pressure, mmHg	140·00 (129·00–157·00)	141·00 (130·00–158·00)	140·00 (126·00–155·00)	140·00 (128·00–156·00)	141·00 (130·00–158·00)
C-reactive protein, mg/L	1·59 (0·97–3·00)	1·53 (0·94–2·78)	1·89 (1·10–3·78)	1·43 (0·84–2·41)	1·77 (1·09–3·50)
BMI, kg/m^2	25·98 (23·51–28·87)	26·15 (23·69–29·03)	25·40 (23·03–28·34)	25·52 (23·32–28·09)	26·38 (23·68–29·58)
Diabetes mellitus	1779 (6·3)	1411 (6·5)	368 (5·5)	603 (4·9)	1139 (7·3)

Alcohol intake		Dietary adherence	
Low	High	High	Low
n=15,042	n=11,470	n=19,427	n=6,265
60·51 (49·23–71·93)	63·47 (53·94–72·50)	61·64 (51·15–71·85)	61·35 (50·57–72·61)
9336 (62·1)	4087 (35·6)	10900 (56·1)	2413 (38·5)
3·00 (0·00–21·75)	12·21 (0·00–32·00)	3·75 (0·00–21·00)	18·75 (0·00–39·00)
9758 (64·9)	6807 (59·3)	11279 (58·1)	4752 (75·8)
1558 (10·4)	1386 (12·1)	2178 (11·2)	562 (9·0)
5·60 (4·90–6·30)	5·70 (5·00–6·50)	5·60 (4·90–6·40)	5·70 (5·00–6·50)
216·55 (189·48–243·62)	220·42 (193·35–251·35)	216·55 (189·48–247·49)	220·42 (193·35–251·35)
3·99 (3·28–4·77)	4·01 (3·29–4·80)	3·96 (3·27–4·74)	4·11 (3·38–4·94)
154·29 (126·84–184·46)	155·07 (127·22–185·62)	153·13 (126·45–183·30)	158·93 (130·70–191·03)
0·66 (0·46–0·96)	0·69 (0·48–1·02)	0·65 (0·45–0·95)	0·74 (0·51–1·09)
25·52 (17·79–37·12)	26·68 (18·56–39·44)	25·14 (17·40–36·74)	28·62 (19·72–42·15)
3·20 (2·60–3·90)	3·21 (2·60–3·90)	3·20 (2·60–3·90)	3·30 (2·70–4·00)
123·74 (100·54–150·81)	124·13 (100·54–150·81)	123·74 (100·54–150·81)	127·61 (104·41–154·68)
1·52 (1·21–1·89)	1·60 (1·28–2·00)	1·58 (1·26–1·95)	1·47 (1·17–1·85)
58·78 (46·79–73·09)	61·87 (49·50–77·34)	61·10 (48·72–75·41)	56·84 (45·24–71·54)
9·98 (4·96–29·67)	9·69 (4·61–28·75)	10·10 (4·96–30·50)	9·28 (4·52–25·97)
17·93 (6·98–60·86)	17·30 (6·23–58·84)	18·19 (6·98–62·66)	16·41 (6·03–52·80)
140·00 (125·00–155·00)	144·00 (130·00–160·00)	140·00 (128·00–157·00)	140·00 (130·00–156·00)
1·57 (0·94–2·98)	1·58 (0·99–2·91)	1·53 (0·93–2·77)	1·80 (1·06–3·67)
25·91 (23·36–28·99)	26·01 (23·68–28·59)	25·89 (23·48–28·75)	26·14 (23·57–29·10)
927 (6·2)	698 (6·1)	1246 (6·4)	315 (5·0)

Values are median (interquartile range) for continuous variables and number of individuals (%) for categorial variables. Baseline characteristics are based on information from the date of examination. Number of individuals vary slightly for individual covariates dependent on availability of covariate. Abbreviations: BMI = body mass index; HDL = high-density lipoprotein; LDL = low-density lipoproteins; NMR = nuclear magnetic resonance spectroscopy.

Figure S1: Proportional Venn Diagram showing percent of individuals with each lifestyle factor

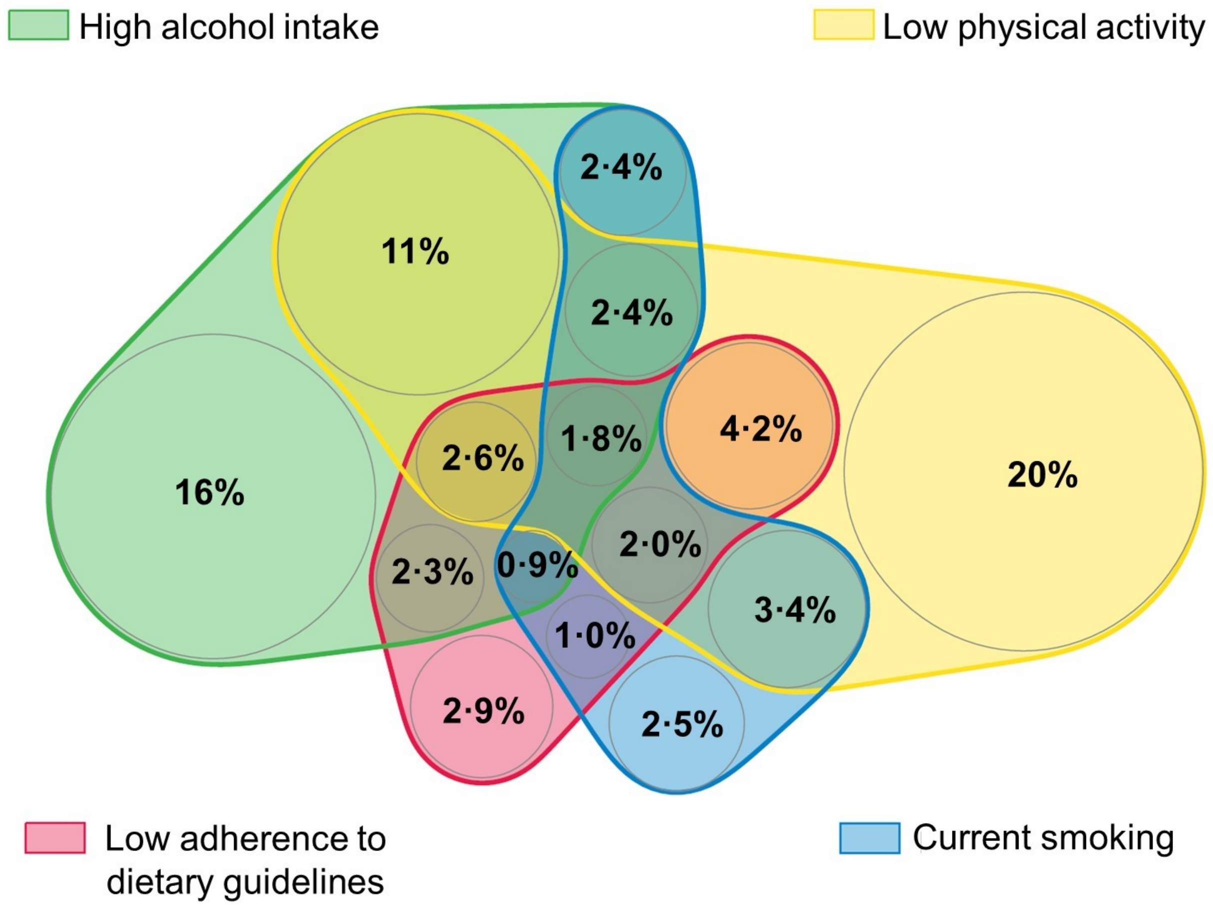

A total of 91,616 individuals with complete information on smoking status, physical activity, alcohol intake, and dietary adherence from the Copenhagen General Population Study were included in this analysis. Only individuals with one or more unhealthy lifestyle factors are shown.

Figure S2: Proportional Venn Diagram showing number of individuals with each lifestyle factor.

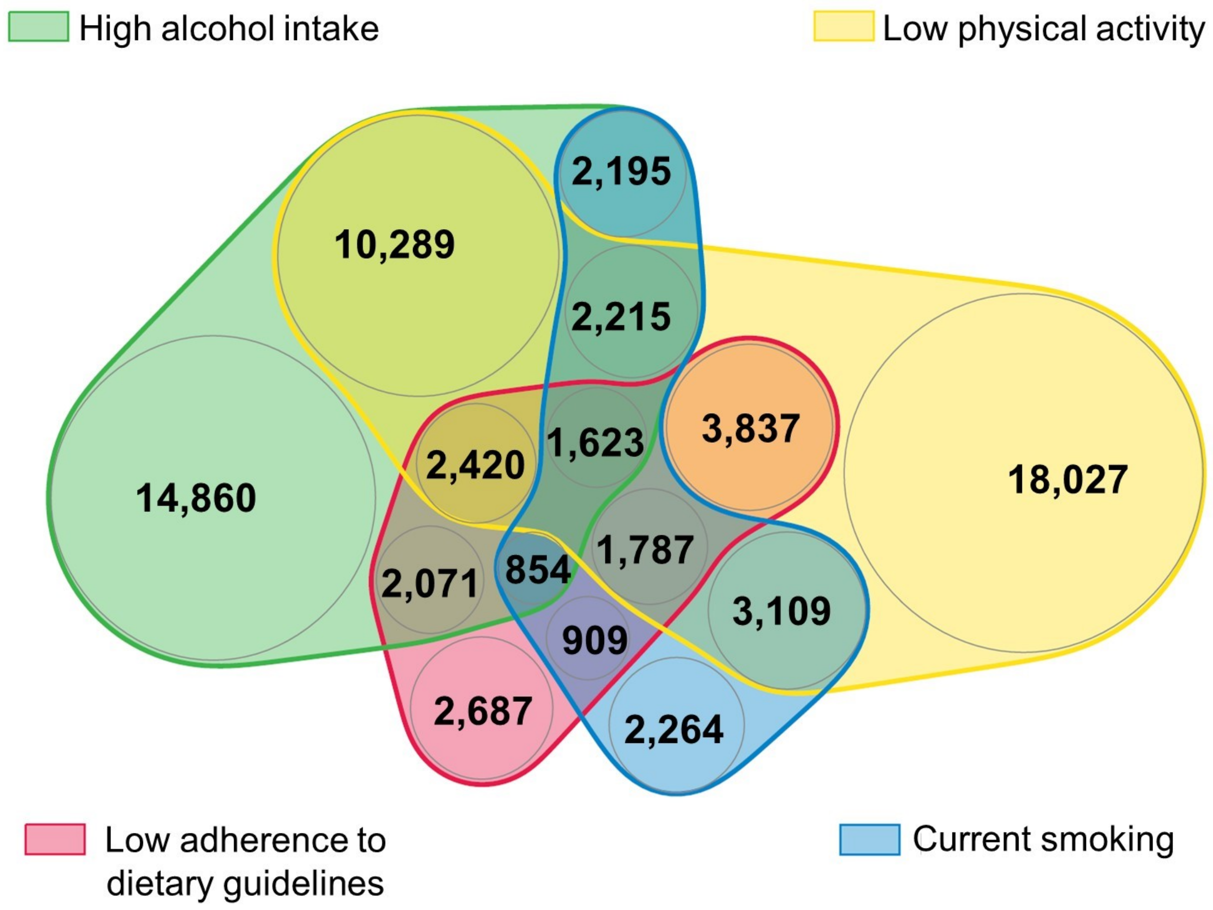

A total of 91,616 individuals with complete information on smoking status, physical activity, alcohol intake, and dietary adherence from the Copenhagen General Population Study were included in this analysis. Only individuals with one or more unhealthy lifestyle factors are shown.

Figure S3: Risk of myocardial infarction, coronary heart disease, and all-cause mortality by exposure variables on continuous scale.

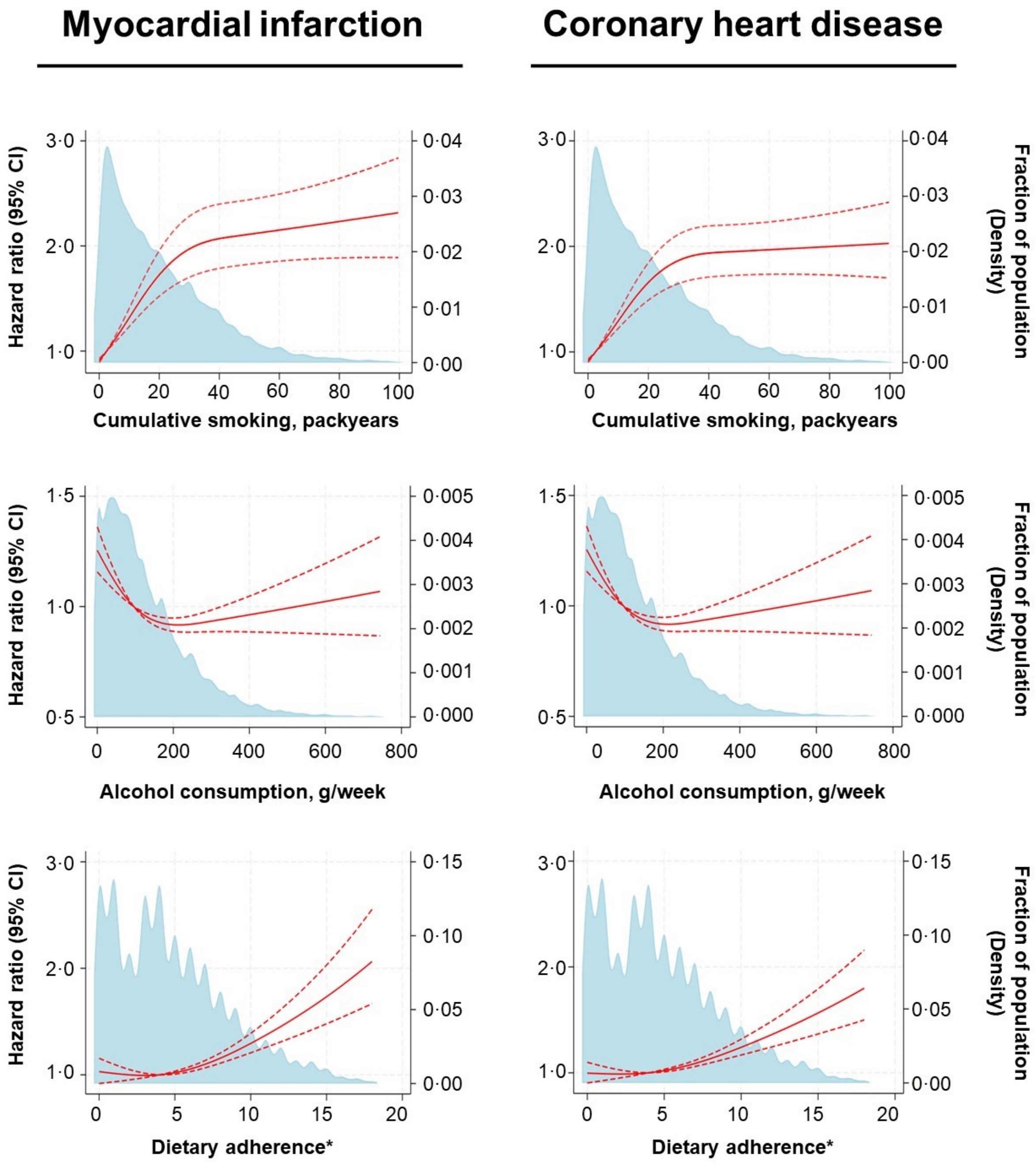

A total of 104,867 individuals from the Copenhagen General Population Study were included in these analyses. During a median follow-up of 9·2 years (ranging from 0–15 years), 2,484 individuals developed myocardial infarction and 3,570 coronary heart disease. Hazard ratios (solid red lines) and 95% confidence intervals (dotted red lines) were multivariable adjusted for age (underlying timescale), sex, and educational level. Fraction of population is shown as density plots (blue) using kernel density estimation. LDL: low-density lipoproteins.

* A value for dietary adherence on a continuous scale was calculated as 3 times the value of class A answer plus 2 times the value of class B answer plus the value of class C answer, as done previously[3].

Figure S4: Risk of myocardial infarction and coronary heart disease according to smoking status, physical activity, alcohol intake, and dietary adherence with each lifestyle factor additionally adjusted for the three other lifestyle factors.

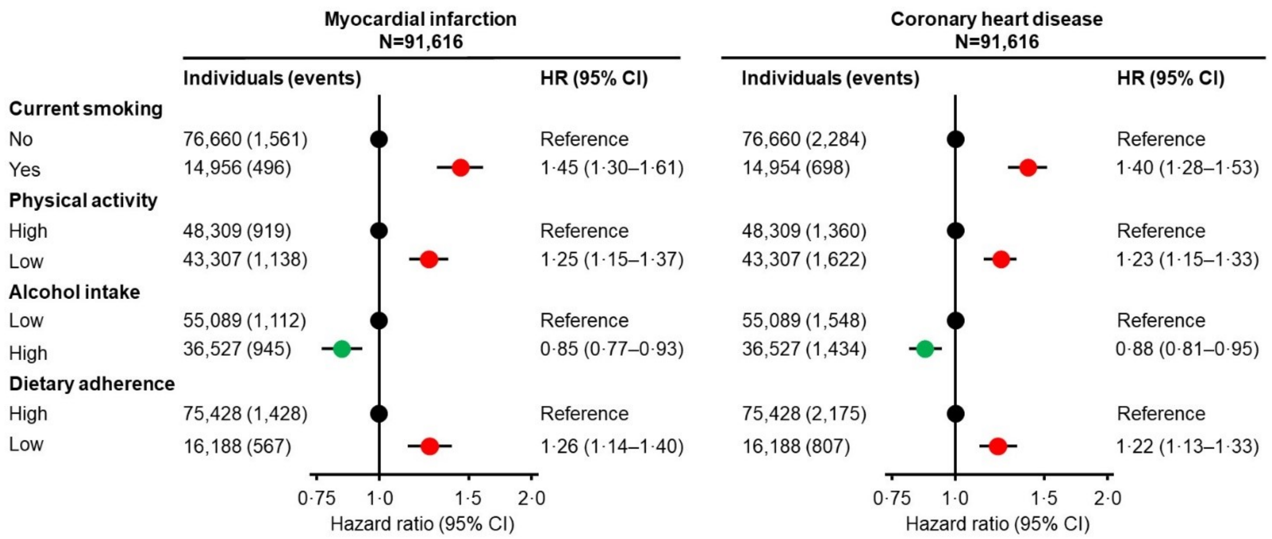

A total of 91,616 individuals from the Copenhagen General Population Study with complete information on smoking status, physical activity, alcohol intake, and dietary adherence were included in the analyses. During a median follow-up of 9·0 years (ranging from 0–15 years), 2,057 individuals developed myocardial infarction and 2,982 developed coronary heart disease. Hazard ratios were multivariable adjusted for age (underlying timescale), sex, and educational level. HR = hazard ratio. CI = confidence intervals.

Figure S5: NMR lipid measurements according to unhealthy lifestyle in women and men, separately.

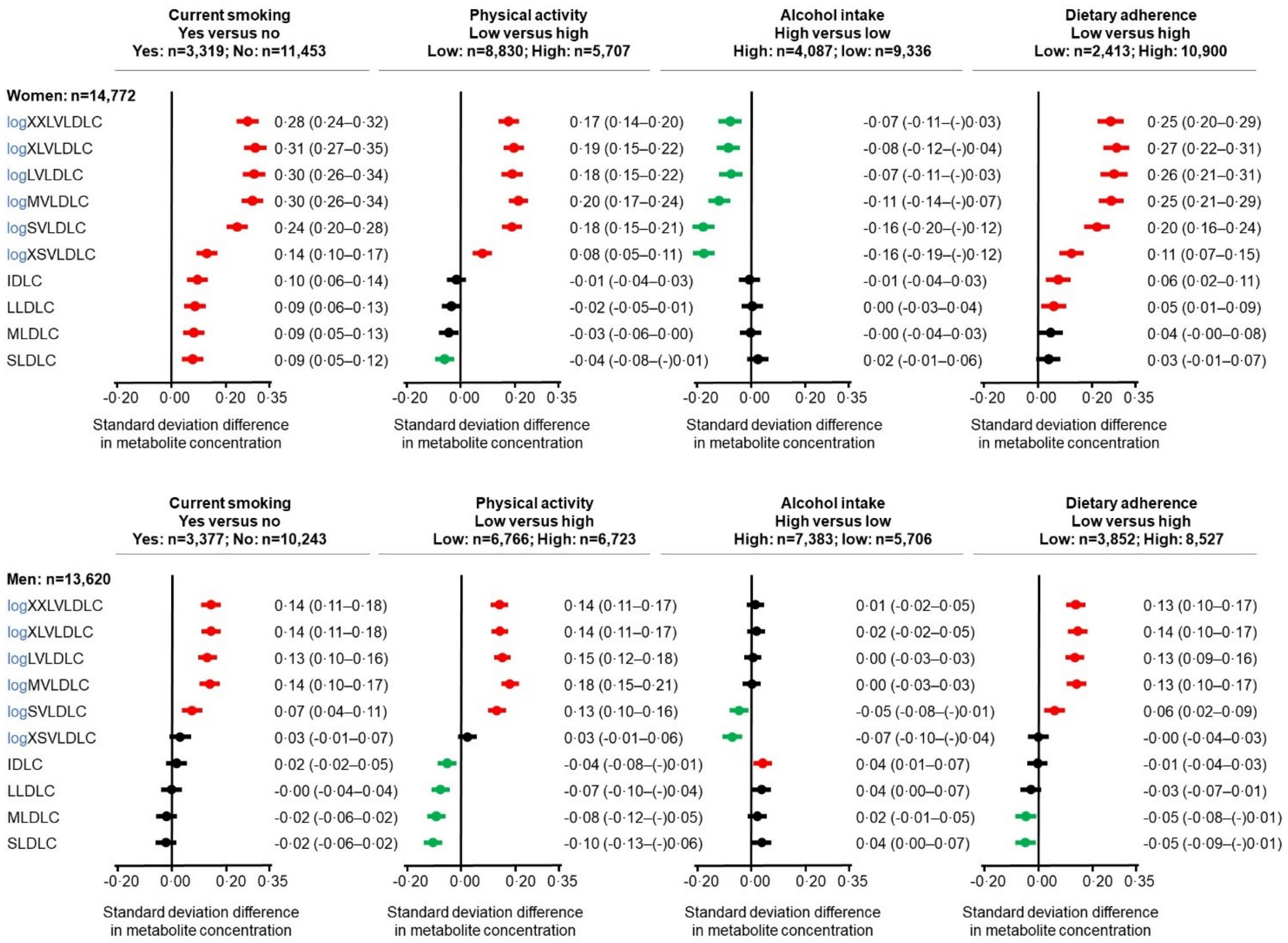

A total of 14,772 women and 13,620 men nested within the Copenhagen General Population Study with NMR measurements available were included in the analyses. Lipid subfractions were estimated by NMR spectroscopy. Skewed variables were logarithmically (log) transformed. Standard deviation differences between groups are shown with 95% CI. Estimates were adjusted for age, sex, and educational level. VLDL = Very low-density lipoproteins. IDL = intermediate-density lipoproteins. LDL = low-density lipoproteins. XXL = extra extra large. XL = extra large. L = large. M = medium. S = small. XS = extra small. NMR = nuclear magnetic resonance. CI = confidence interval.

Figure S6: Explained excess risk from unhealthy lifestyle with each lifestyle factor additionally adjusted for the other three lifestyle factors

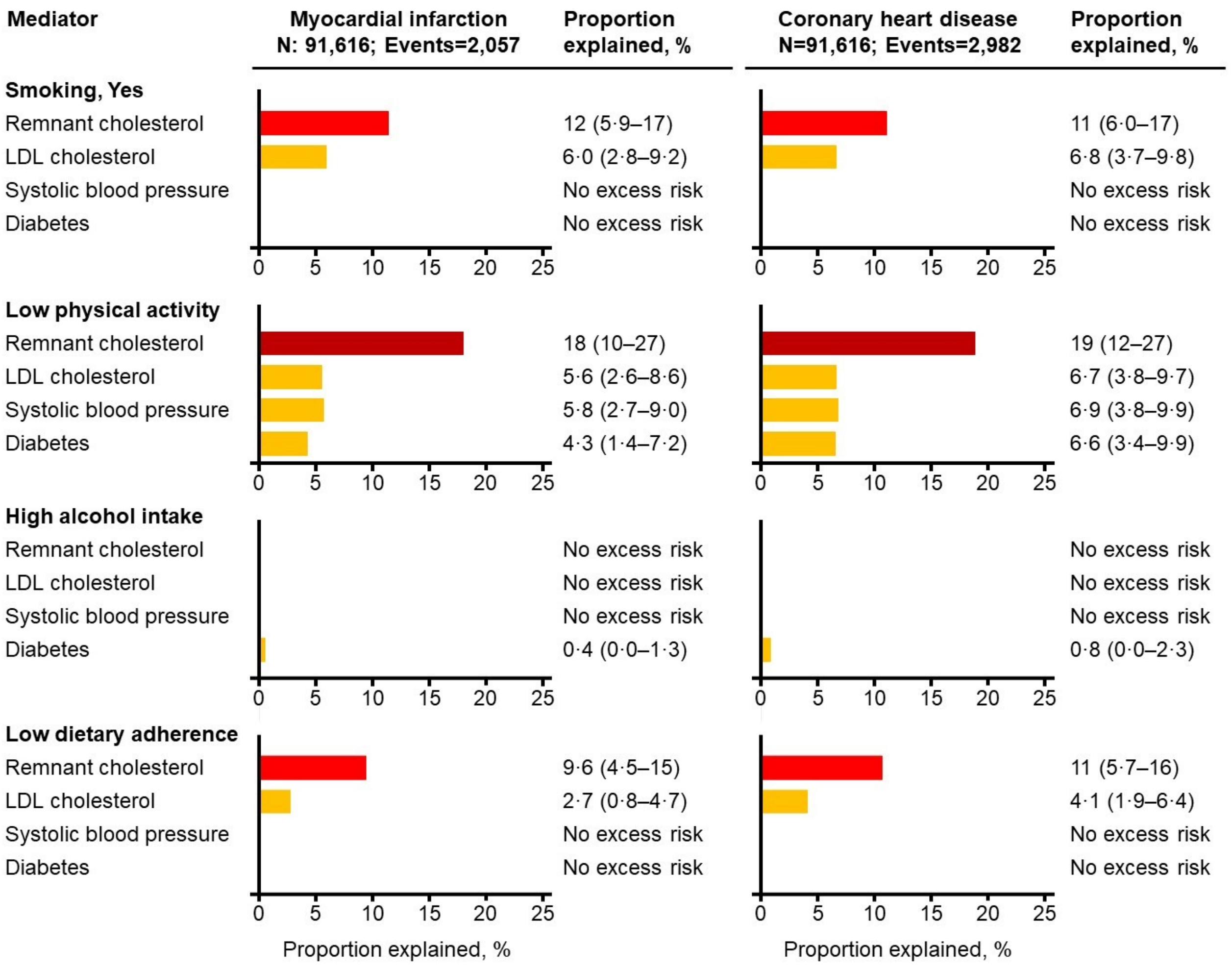

Percent excess risk of myocardial infarction and coronary heart disease from unhealthy lifestyle explained by intermediate variables in 91,616 individuals from the Copenhagen General Population Study with complete information on smoking status, physical activity, alcohol intake, and dietary adherence using the method by VanderWeele[1]. During a median follow-up of 9·0 years (ranging from 0–15 years), 2,057 individuals developed myocardial infarction and 2,982 developed coronary heart disease. Estimated excess risk in percent with 95% CIs for each exposure-mediator-outcome relationship are shown. As possible explanatory factors, we used elevated remnant cholesterol, elevated LDL cholesterol, elevated systolic blood pressure, and diabetes mellitus, because these are known causal factors for increased risk of myocardial infarction and coronary heart disease. Estimates were multivariable adjusted for age (underlying time scale), sex, and educational level, and truncated at zero excess risk explained. LDL = low-density lipoprotein.

Figure S7: Explained excess risk in unhealthy lifestyle **__using the product of coefficients method__**

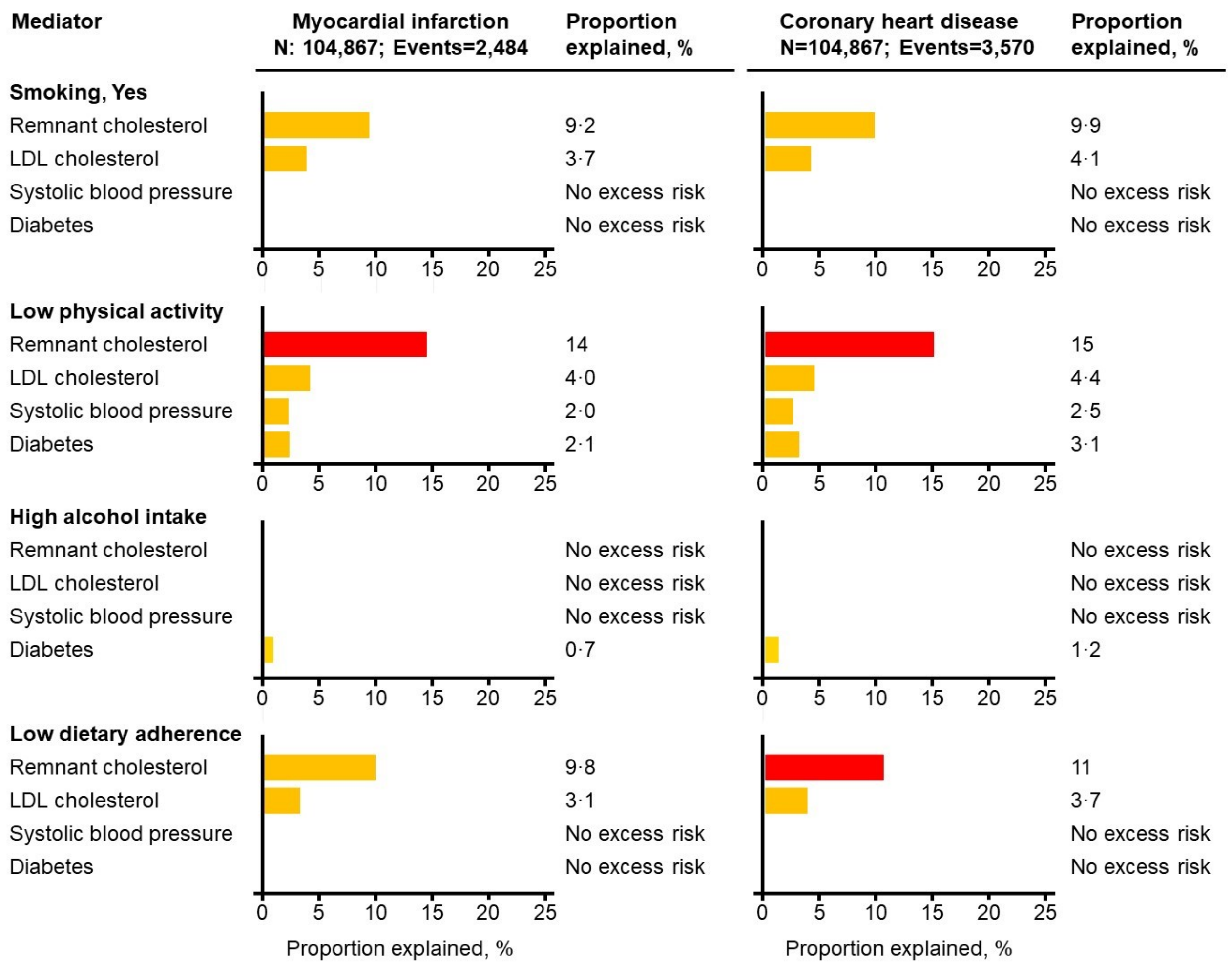

Percent excess risk of myocardial infarction and coronary heart disease from unhealthy lifestyle explained by intermediate variables in 104,867 individuals from the Copenhagen General Population Study using the product of coefficient method[2]. During a median follow-up of 9·2 years (ranging from 0–15 years), 2,484 individuals developed myocardial infarction and 3,570 coronary heart disease. Mediation analyses were conducted using the product of coefficients method[2]. Estimated mediated proportion in percent for each exposure-mediator-outcome relationship are shown. As possible explanatory factors, we used elevated remnant cholesterol, elevated LDL cholesterol, elevated systolic blood pressure, and diabetes mellitus, because these are known causal factors for increased risk of myocardial infarction and coronary heart disease. Estimates were multivariable adjusted for age (underlying time scale), sex, and educational level, and truncated at zero excess risk explained. LDL = low-density lipoprotein.

Figure S8: Explained excess risk in unhealthy lifestyle <u>using different definitions of smoking status</u>

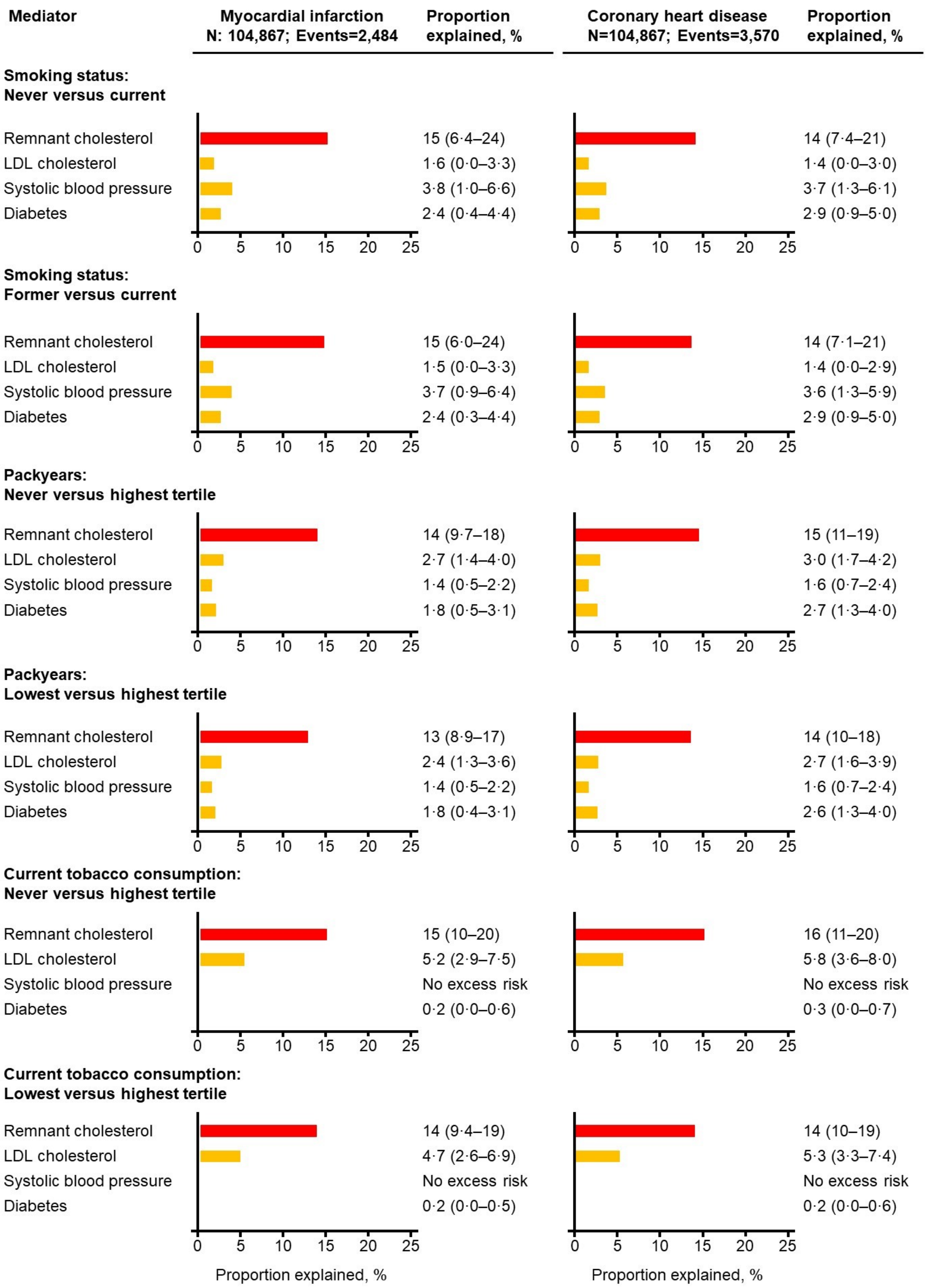

Percent excess risk of myocardial infarction and coronary heart disease from smoking status explained by intermediate variables in 104,867 individuals from the Copenhagen General Population Study using the method by VanderWeele[1]. During a median follow-up of 9·2 years (ranging from 0–15 years), 2,484 individuals developed myocardial infarction

and 3,570 individuals developed coronary heart disease. Estimated mediated proportion in percent for each exposure-mediator-outcome relationship are shown. As possible explanatory factors, we used elevated remnant cholesterol, elevated LDL cholesterol, elevated systolic blood pressure, and diabetes mellitus, because these are known causal factors for increased risk of myocardial infarction and coronary heart disease. Estimates were multivariable adjusted for age (underlying time scale), sex, and educational level, and truncated at zero excess risk explained. LDL = low-density lipoprotein.

Figure S9: Risk of myocardial infarction, coronary heart disease, and all-cause mortality by intermediate variables on continuous scale.

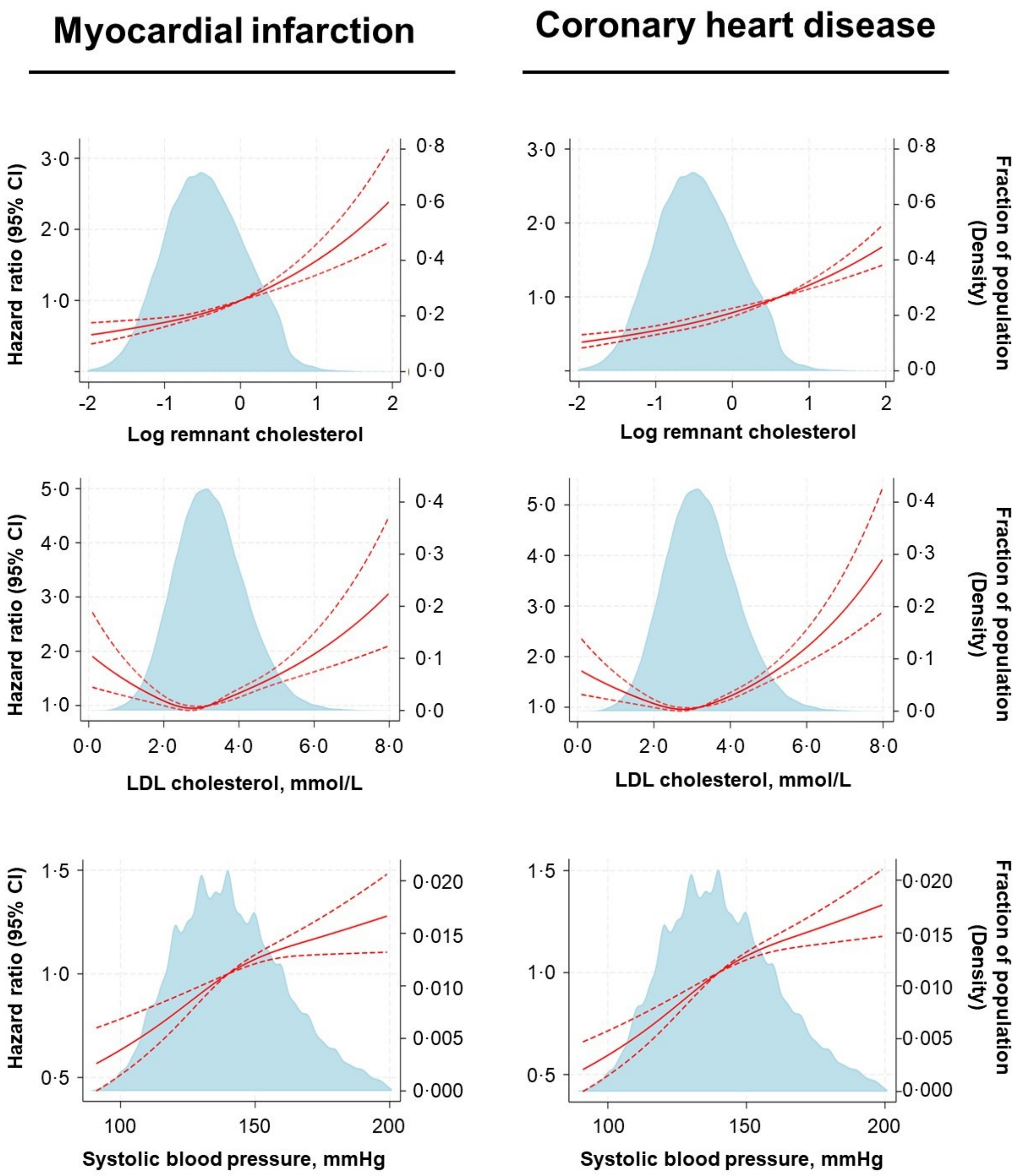

A total of 104,867 individuals from the Copenhagen General Population Study were included in these analyses. During a median follow-up of 9·2 years (ranging from 0–15 years), 2,484 individuals developed myocardial infarction and 3,570 coronary heart disease. Hazard ratios (solid red lines) and 95% confidence intervals (dotted red lines) were multivariable adjusted for age (underlying timescale), sex, and educational level. Fraction of population is shown as density plots (blue) using kernel density estimation. LDL: low-density lipoproteins.

Figure S10: Explained excess risk in unhealthy lifestyle in individuals with type 2 diabetes

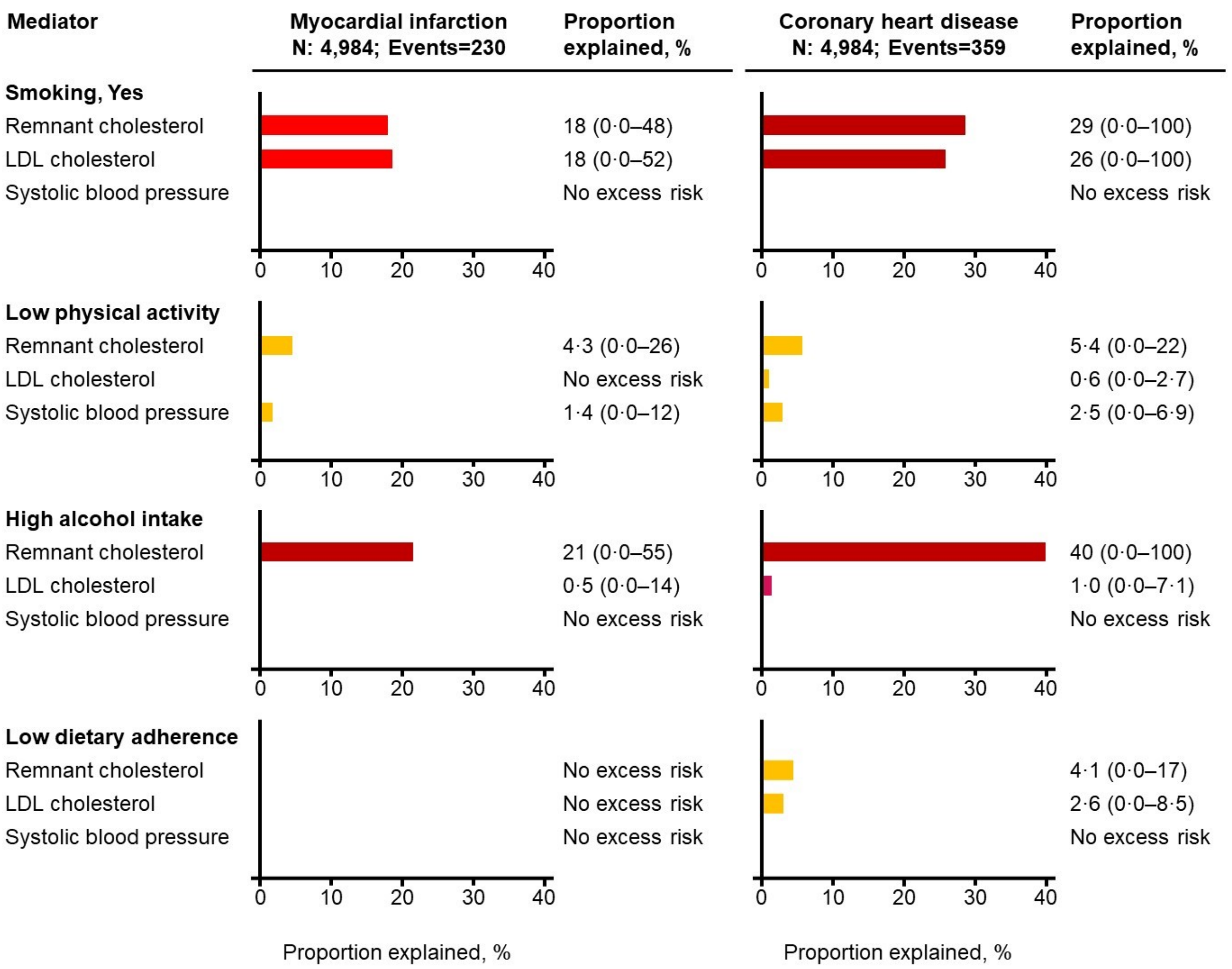

Percent excess risk of myocardial infarction and coronary heart disease from smoking status explained by intermediate variables in 4,984 individuals with type 2 diabetes from the Copenhagen General Population Study using the method by VanderWeele[1]. During a median follow-up of 7·6 years (ranging from 0–15 years), 230 individuals developed myocardial infarction and 359 coronary heart disease. Estimated mediated proportion in percent for each exposure-mediator-outcome relationship are shown. As possible explanatory factors, we used elevated remnant cholesterol, elevated LDL cholesterol, and elevated systolic blood pressure, because these are known causal factors for increased risk of myocardial infarction and coronary heart disease. Estimates were multivariable adjusted for age (underlying time scale), sex, and educational level, and truncated at zero excess risk explained. LDL = low-density lipoprotein.

References

1. VanderWeele TJ. Mediation Analysis: A Practitioner's Guide. *Annu Rev Public Health* 2016; **37**: 17-32.
2. MacKinnon DP, Fairchild AJ, Fritz MS. Mediation analysis. *Annu Rev Psychol* 2007; **58**: 593-614.
3. Ewers B, Marott JL, Schnohr P, Nordestgaard BG, Marckmann P. Non-adherence to established dietary guidelines associated with increased mortality: the Copenhagen General Population Study. *Eur J Prev Cardiol* 2021; **28**(11): 1259-68.
